# Low energy carbon capture via electrochemically induced pH swing with electrochemical rebalancing

**DOI:** 10.1038/s41467-022-29791-7

**Published:** 2022-04-19

**Authors:** Shijian Jin, Min Wu, Yan Jing, Roy G. Gordon, Michael J. Aziz

**Affiliations:** 1 John A. Paulson School of Engineering and Applied Sciences, Harvard University, Cambridge, MA 02138 USA; 2 Department of Chemistry and Chemical Biology, Harvard University, Cambridge, MA 02138 USA

**Keywords:** Carbon capture and storage, Chemical engineering, Chemistry

## Abstract

We demonstrate a carbon capture system based on pH swing cycles driven through proton-coupled electron transfer of sodium (3,3′-(phenazine-2,3-diylbis(oxy))bis(propane-1-sulfonate)) (DSPZ) molecules. Electrochemical reduction of DSPZ causes an increase of hydroxide concentration, which absorbs CO_2_; subsequent electrochemical oxidation of the reduced DSPZ consumes the hydroxide, causing CO_2_ outgassing. The measured electrical work of separating CO_2_ from a binary mixture with N_2_, at CO_2_ inlet partial pressures ranging from 0.1 to 0.5 bar, and releasing to a pure CO_2_ exit stream at 1.0 bar, was measured for electrical current densities of 20–150 mA cm^−2^. The work for separating CO_2_ from a 0.1 bar inlet and concentrating into a 1 bar exit is 61.3 kJ mol_CO2_^−1^ at a current density of 20 mA cm^−2^. Depending on the initial composition of the electrolyte, the molar cycle work for capture from 0.4 mbar extrapolates to 121–237 kJ mol_CO2_^−1^ at 20 mA cm^−2^. We also introduce an electrochemical rebalancing method that extends cell lifetime by recovering the initial electrolyte composition after it is perturbed by side reactions. We discuss the implications of these results for future low-energy electrochemical carbon capture devices.

## Abstract (web summary)

This work demonstrates a safe and scalable electrochemical CO_2_ separation method that allows promisingly low (62 kJ/mol_CO2_) energetic cost at a high current density, and it can be used for direct air capture when a suitable molecule is used.

## Introduction

Accumulating CO_2_ emissions from anthropogenic activities constitute the major cause of global climate change^1,2^. While efforts are being made in switching from fossil fuel-based energy to virtually emissions-free sources such as nuclear, solar, wind and geothermal, fossil fuel combustion will remain an important component of the world economy for a long time^3^. Consequently, carbon removal—whether captured from a point source^2,4–8^ such as a combustion power plant or directly from the air (a.k.a. direct air capture, DAC) or the ocean^2,9–13^—in order to reduce atmospheric CO_2_ concentrations, is gaining increasing attention.

Numerous methods for point source capture and DAC have been developed. Among the most studied is wet amine scrubbing for point source capture^4–6^ and strongly alkaline (pH > 14) solution for DAC^2,9^, both of which rely on a large temperature-swing cycle to regenerate sorbents. Although sorbent composition has been optimized to lower the energy cost for both strategies, the thermal energy requirement for heating is still ~100 kJ mol_CO2_^−1^ for point source capture^6,14,15^ and >150 kJ mol_CO2_^−1^ for DAC^10,16^. In addition, sorbent volatility, toxicity, and corrosivity cause environmental concerns^2^. Methods that remove CO_2_ from the ocean, which allow it to absorb more CO_2_, have also been studied, but the high water-handling requirement is a challenge^12,13^.

Electrochemically mediated separation technologies constitute an increasingly attractive alternative to traditional temperature-swing or pressure-swing methods because of the rapidly decreasing cost of intermittent renewable electricity and the mild operating conditions of ambient temperature and pressure^7,8,13,17–20^. However, most existing methods operate at low current density (<5 mA cm^−2^) because of large overpotentials and the corresponding energetic cost at higher current density, implying a high capital cost of electrochemical hardware. Recently, our group proposed and demonstrated a pH swing cycle for CO_2_ separation electrochemically driven through proton-coupled electron transfer (PCET) of redox active organic molecules (“Q”)^18^. In this scheme, proton-coupled electrochemical reduction of these molecules (Q + 2H_2_O + 2*e*^−^ → QH_2_ + 2OH^−^) raises the electrolyte pH and total alkalinity (TA), leading to CO_2_ capture from point source or air and conversion to dissolved inorganic carbon (DIC); subsequent electrochemical oxidation of the reduced molecules (QH_2_ + 2OH^−^ → Q + 2H_2_O + 2*e*^−^) acidifies the electrolyte and lowers TA, resulting in the conversion of DIC to CO_2_ gas and its release.

**Fig. 1:**
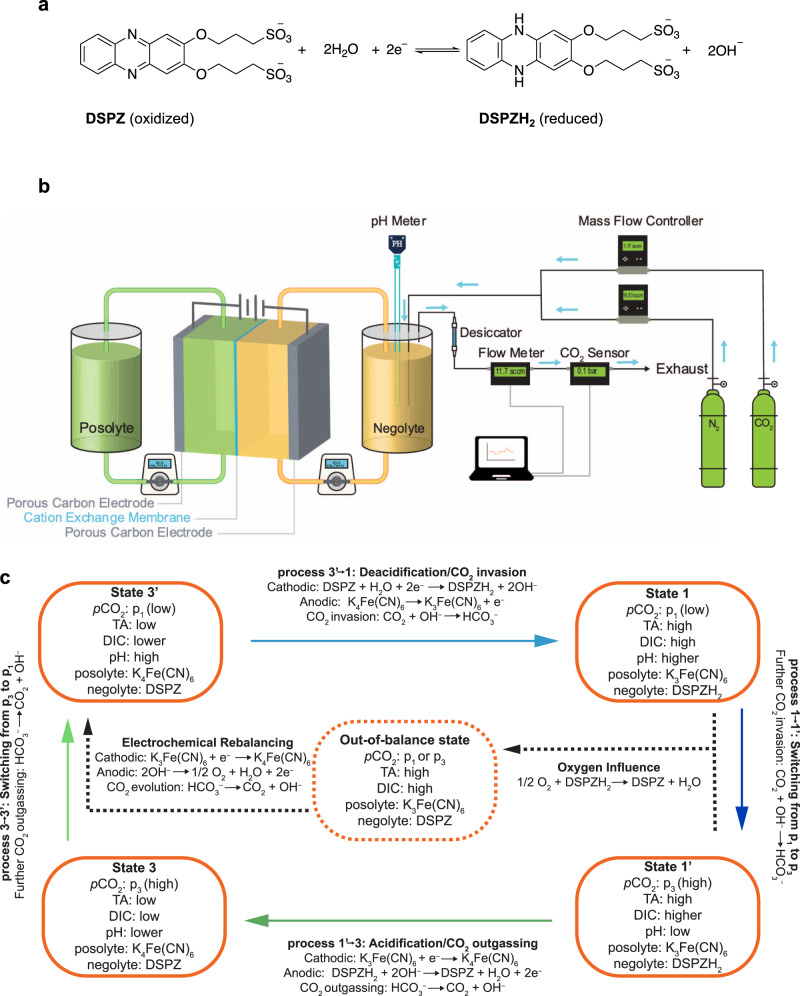
Scheme of the pH swing carbon capture flow system. **a** Schematic of the reversible PCET (proton-coupled electron transfer) reaction underwent by DSPZ (sodium (3,3′-(phenazine-2,3-diylbis(oxy))bis(propane-1-sulfonate))) in an aqueous solution. **b** Schematic of the Fe(CN)_6_ (posolyte) | DSPZ (negolyte) flow cell and full system. Blue arrows indicate gas flow direction. Adapted from ref. ^18^. **c** Process flow. TA is total alkalinity and DIC is dissolved inorganic carbon. The solid arrows refer to desired reactions in a complete carbon capture/release cycle. The carbonate formation and decomposition reactions are neglected for simplicity. The dashed arrow on the right side refers to the side reaction caused by oxygen and the dashed arrow on the left refers to reactions in the electrochemical rebalancing step.

Here, we report a proof-of-concept point source (10%) CO_2_ separation system that uses a sodium (3,3′-(phenazine-2,3-diylbis(oxy))bis(propane-1-sulfonate)), i.e., DSPZ (Fig. 1a), based electrochemical pH-swing cell with an energy cost of only 61.3 kJ mol_CO2_^−1^ at 20 mA cm^−2^. Through analyzing the cycle work obtained under systematically varied inlet partial pressure and current density, we estimate that the cost for capturing from a 0.4 mbar CO_2_ inlet using this system extrapolates to 121– 237 kJ mol_CO2_^−1^ at 20 mA cm^−2^, and that it can be further lowered if a higher concentration of DSPZ, or other PCET-active molecules, is used. Recognizing the sensitivity of the reduced form of DSPZ, i.e., DSPZH_2_, to chemical oxidation by atmospheric or dissolved O_2_, we introduce and demonstrate an electrochemical rebalancing method that expels oxygen from solution and restores the initial composition of the electrolytes.

## Results and discussion

### Device setup and process flow

Figure 1b shows the schematic of the Fe(CN)_6_|DSPZ carbon capture flow cell and the hardware for providing the gas mixture and analyzing the exhaust. The upstream gas composition in the negolyte headspace was controlled by CO_2_ and N_2_ mass flow controllers (MFCs). Downstream of the negolyte reservoir, the gas was dried with a desiccator and the total gas flow rate and CO_2_ partial pressure were measured using a digital flow meter and a CO_2_ sensor, respectively. A pH probe immersed in the negolyte solution reported the temporal evolution of its pH, which enabled the tracking of total alkalinity (TA) and dissolved inorganic carbon (DIC) in real time. Figure 1c illustrates the electrolyte composition in the four states of the pH swing carbon capture cycle and the processes connecting the states. We denote the CO_2_ partial pressure during the CO_2_ capture process as the inlet pressure or *p*_1_, and that during the CO_2_ outgassing process as the exit pressure or *p*_3_, which is always set to 1 bar (100% CO_2_) in this study. Similarly, the subscripts following TA or DIC refer to the TA and DIC of the corresponding states. The naming convention for the states is adopted from previous work^18^, where the equilibrium and constraints governing pH, TA, DIC, and *p*CO_2_ are explained in detail.

The four colored arrows in Fig. 1c refer to the four processes in the carbon capture cycle. The four sequential processes are as follows: 3′_i_→1: two-stage deacidification+CO_2_ invasion (inlet). In this process, DSPZ is electrochemically reduced to DSPZH_2_, and hydroxide is produced, which reacts with CO_2_ to form carbonate/bicarbonate. 1→1′: change of the headspace atmosphere from inlet to exit pressure, i.e., switching from *p*_1_ to *p*_3_. 1′→3: two-stage acidification+CO_2_ outgassing (exit). In this process DSPZH_2_ is electrochemically oxidized to DSPZ and hydroxide is consumed, which in turn leads to carbonate/bicarbonate decomposition and CO_2_ evolution. 3→3′_f_: change of the headspace atmosphere from exit to inlet pressure, i.e., switching from *p*_3_ to *p*_1_. The change in TA and DIC in these processes are denoted with ΔTA_a→b_ and ΔDIC_a→b_, respectively, where the subscript refers to process “a→b”, and a and b are any pairs of states. An example cycle is described quantitatively in the next section.

As DSPZH_2_ is reversibly chemically oxidized by atmospheric O_2_ to DSPZ (right dashed arrow in Fig. 1c), the posolyte supplies extra charge to electrochemically reduce the extra oxidized DSPZ; this is reflected in the low Coulombic efficiency of the cell and an accumulation of TA and DIC in the negolyte. This process also transforms more of the posolyte to its oxidized form, i.e., [K^+^]_4_[Fe^II^(CN)_6_]^4−^→ [K^+^]_3_[Fe^III^(CN)_6_]^3−^+ K^+^ + *e*^−^, than at a similar point in the previous deacidification-acidification cycle. During cell operation reduction on one side must be accompanied by oxidation on the other side but, as the available fraction of reduced species on the posolyte side, i.e., [K^+^]_4_[Fe^II^(CN)_6_]^4−^, decreases, the cell can access less and less of its theoretical capacity during its oxidation-reduction oscillations; this is reflected in the decaying capacity of the cell. Eventually, both sides become 100% oxidized and cell operation ceases. Our remedy for such situations is the electrochemical rebalancing method (left dashed arrow in Fig. 1c) explained later in the text.

### One carbon capture cycle with *p*_1_ = 0.1 bar and *p*_3_ = 1 bar at 40 mA cm^−2^

**Fig. 2:**
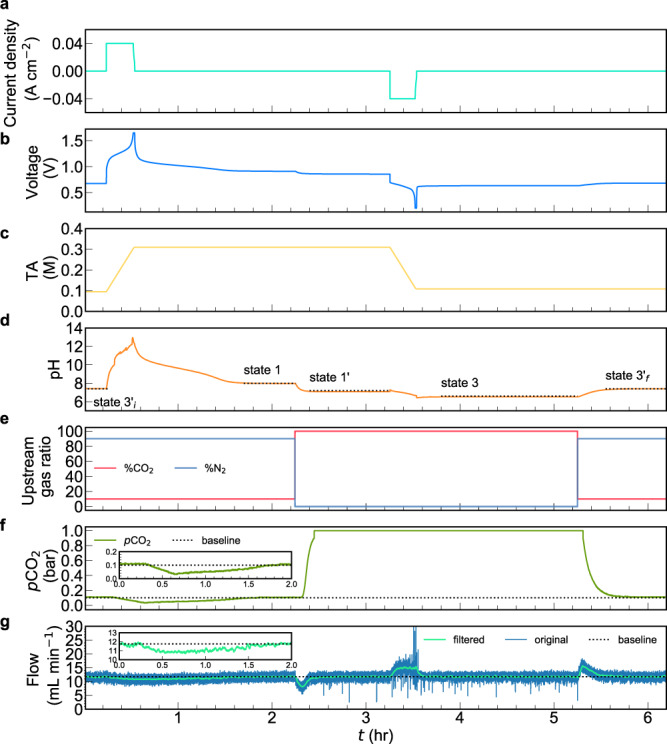
A CO_2_ concentrating cycle with inlet pressure *p*_1_ = 0.1 bar and exit pressure *p*_3_ = 1 bar using a DSPZ-based flow cell at 40 mA cm^−2^. Electrolytes comprised 10 mL 0.11 M DSPZ in 1 M KCl (negolyte, capacity limiting) and 35 mL 0.1 M K_4_Fe(CN)_6_ and 0.04 M K_3_Fe(CN)_6_ in 1 M KCl (posolyte, non-capacity limiting). **a** Current density. **b** Voltage. **c** Total alkalinity. **d** pH of the negolyte. States 3′_i_, 1, 1′, 3 and 3′_f_ represent pH values before deacidification under 0.1 bar *p*CO_2_, after deacidification+absorption under 0.1 bar *p*CO_2_, after changing *p*CO_2_ from 0.1 bar to 1 bar, after acidification+desorption under 1 bar and after changing *p*CO_2_ from 1 bar to 0.1 bar, respectively. The detailed composition of these states is elaborated in Table 1. **e** N_2_ and CO_2_ percentage in the upstream source gas, controlled by mass flow controllers. **f** Downstream CO_2_ partial pressure. The baseline indicates *p*CO_2_ = 0.1 bar. Inset: Zoomed-in view of downstream CO_2_ partial pressure in between 0 < *t* < 2 h, where CO_2_ capture takes place. **g** Downstream total gas flow rate; the baseline is 11.8 mL min^−1^. Inset: Zoomed-in view of downstream gas flow rate (filtered) in between 0 < *t* < 2 h, where CO_2_ capture takes place.

In previous work we demonstrated a series of non-concentrating cycles, in which both exit and inlet *p*CO_2_ were 0.47 bar, utilizing a DSPZ-based flow cell at 40−150 mA cm^−2^^18^. In the present work, we show the use of this setup for CO_2_ separation from low partial pressure in a mixture with nitrogen and release into a pure CO_2_ exit stream at 1.0 bar. Figure 2 demonstrates one such cycle with *p*CO_2_ = 1.0 and 0.1 bar at the exit and inlet, respectively. Beginning at state 3′_i_, the upstream CO_2_ partial pressure was set to 0.1 bar, which is close to its value in the flue gas from coal fired power plants^7^. We define *t* as the time elapsed. As deacidification began (Fig. 2a, b, *t* = 0.2 h), the TA went up at a linear rate because only K^+^ ions crossed the cation exchange membrane (CEM) when a constant 40 mA cm^−2^ current density was applied (Fig. 2c)^18^. As a result of the PCET reactions during the reduction of DSPZ, the negolyte pH (Fig. 2d) increased from near neutral to ~13.5 at the end of the deacidification process, indicated by the steep increase of voltage until reaching the preset voltage cutoff of 1.65 V (Fig. 2b, *t* = 0.6 h). CO_2_ invasion began when deacidification began but continued beyond the end of deacidification: invasion lasted until *t* = 1.8 h, as indicated by the *p*CO_2_ signal returning to the 0.1 bar baseline, because of the limited reaction rate between dilute OH^−^ and CO_2_. The deviation in the gas flow rate (Fig. 2g) from the baseline starting at *t* = 0.2 h and returning at 1.8 h also documents the complete capture process. As CO_2_ reacted with hydroxide and water to form CO_3_^2−^ and HCO_3_^−^, the pH (Fig. 2d) dropped from ~13.5 at *t* = 0.5 h to 8.1 at 1.8 h and then plateaued, once again indicating the completion of the capture process. The absorbed volume of CO_2_ is 47 mL (Eq. (1) in “Methods”). Assuming *T* = 293 K, *p* = 1 bar, and ideal gas behavior, this absorption causes a change in DIC of 0.20 M (2.0 mmol CO_2_ in 10 mL negolyte volume). We denote this change as ΔDIC_flow,3_′_i→1_, where the subscript “*flow*” indicates that the value is measured by the downstream flowmeter and CO_2_ sensor and “*3*′*i*→*1”* indicates that this value corresponds to the change in process 3′_i_→1 (Table 1). The same naming convention is used for both ΔTA and ΔDIC throughout the rest of this text. Unlike the flowmeter and CO_2_ sensor, which quantify ΔDIC, the pH probe, in addition to providing a measured value (pH_meas_), provides a direct measurement of DIC, because given two values from TA, DIC, *p*CO_2_ and pH, the others can be derived^18,21^. At state 3′, the DIC (regardless of subscripts) and TA values are calculated using pH_meas_ and assumed gas-solution equilibrium, i.e., CO_2_(*aq*) = 0.035 × *p*CO_2_, where 0.035 comes from Henry’s law constant of 35 mM bar^−1^ for CO_2_ at room temperature. Because ΔTA_3′i→1_ is known from Fig. 2c, TA_1_ can be evaluated, and so can the TA values at other states. One way of obtaining DIC in all states except 3′_i_ and of obtaining ΔDIC values between all states is to use the known TA and CO_2_(aq), and we denote these values with subscript “TA–eq” (Table 1). This method is also used to construct the ideal cycles (Supplementary Fig. 1). Another way to calculate DIC is to use the TA and pH_meas_ without assuming gas-solution equilibrium. We denote DIC and ΔDIC calculated this way with subscript “TA−pH”. The ΔDIC between state 3′_i_ and 1_,_ i.e., 0.20 M, determined by flow meter and CO_2_ sensor, i.e., ΔDIC_flow,3′i→1_, is corroborated by ΔDIC_TA−pH,3′i→1_, and ΔDIC_TA−eq,3′i→1_ (Table 1).

**Table 1:** Summary of TA, *p*CO_2_, pH, DIC and ΔDIC.

State	TA (M)	p_*1*_*,p*CO_2_ (bar)	pH_meas_	pH_TA−eq_	DIC_flow_ (M)	DIC_TA−pH_ (M)	DIC_TA−eq_ (M)	ΔDIC_flow_ (M)	ΔDIC_TA−pH_ (M)	ΔDIC_TA−eq_ (M)
3′_i_	0.11^a^	0.1	7.4	7.4^a^	0.11^a^	0.11^a^	0.11^a^			
								0.20	0.20	0.20
1	0.32	0.1	8.1	7.9	0.31	0.31	0.31			
								N/A	0.03	0.03
1′	0.32	1.0	7.1	6.9	0.34^b^	0.34	0.34			
								0.20	−0.20	−0.20
3	0.12	1.0	6.6	6.5	0.14	0.14	0.14			
								N/A	−0.03	−0.03
3′_f_	0.12	0.1	7.5	7.5	0.12^c^	0.12	0.12			

TA is calculated by counting charges and assuming Κ^+^ is the only ion passing through the CEM; *p*CO_2_ is the CO_2_ partial pressure at each state; pH_meas_ refers to the negolyte pH measured by the pH probe. All DIC and TA values at state 3′_i_ are calculated using the measured pH and assuming gas-solution equilibrium. In all other states, pH_TA−eq_ and DIC_TA−eq_ are calculated using TA and assuming gas-solution equilibrium. DIC_TA−pH_ is calculated using TA and pH_meas_; ΔDIC_TA−pH_ and ΔDIC_TA−eq_ are the difference in DIC_TA−pH_ and DIC_TA−eq_ values, respectively, between two consecutive states; ΔDIC_flow_ is converted from the volume of CO_2_ captured or released, measured by the downstream flow meter and CO_2_ sensor and DIC_flow_ is calculated by adding ΔDIC_flow_ at the current state to DIC_flow_ at the state one row above. Because ΔDIC_flow_ is not measurable between states 1 and 1′ and states 3 and 3′_f_, DIC_flow_ at states 1′ and 3′_f_ is calculated by adding DIC_flow_ with ΔDIC_TA−pH_ values between the corresponding states.

^a^All values derived pH_meas_, assuming gas-solution equilibrium.

^b^Calculated by summing DIC_flow,1_ and ΔDIC_TA−pH,1−1′_.

^c^Calculated by summing DIC_flow,3_ and ΔDIC_TA−pH,3−3′f_.

After CO_2_ capture at 0.1 bar (*p*_1_) was completed, the headspace of the negolyte was switched to a pure CO_2_ environment (*p*_3_) to prepare for CO_2_ outgassing, i.e., going through process 1→1′ (Fig. 2e, *t* = 2.2 h). The drop in flow rate at *t* = 2.2 h and its return to the baseline at 2.5 h is caused by the combined effect of mismatched valve response rate in the MFCs (the N_2_ MFC valve closes faster than the CO_2_ MFC valve opens) and a small increase in DIC due to increased *p*CO_2_ in the headspace. This increase in DIC, corresponding to ΔDIC_1→1′_, is difficult to quantify via the flowmeter and CO_2_ sensor, but can be determined using pH_meas_ (ΔDIC_TA−pH,1→1′_) or assuming gas-solution equilibrium (ΔDIC_TA−eq,1→1′_), which both give 0.03 M (Table 1). The acidification+CO_2_ outgassing (process 1′→3) started at *t* = 3.2 h and ended at a little over 3.6 h (Fig. 2a–d, g). Note that, unlike in process 3′_i_→1, the CO_2_ outgassing, which is observable from pH change and an increase in flow rate, (Fig. 2d, g) lasted for no more than ten minutes after the acidification process (Fig. 2a, b) finished. The outgassed CO_2_ volume was 49 mL (Eq. 1), which is equivalent to ΔDIC_flow,1′__→3_ = −0.20 M. Once again, ΔDIC_TA−pH_ and ΔDIC_TA−eq_ agree with ΔDIC_flow_ for the changes between states 1′ and 3. Starting from a little over *t* = 5.2 h, the headspace was filled with 0.1 bar CO_2_ + 0.9 bar N_2_ to recover the state 3’ for the next cycle (process 3→3′_f_). Like process 1→1′, there was a combined effect of valve response and additional CO_2_ outgassing during process 3→3′_f_, causing an increase in flow rate (Fig. 2g, *t* = 5.2 h to 5.6 h). Note that state 3′_f_ has slightly higher pH and 0.01 M more of TA and DIC than state 3′_i_ because of the influence of oxygen (Fig. 1c).

### Calculation of ΔDIC_flow,3→1_, molar cycle work, and productivity

The discussion above shows how ΔDIC_flow,3′i→1_ and ΔDIC_flow,1′→3_ are obtained by gas flow measurement, but neither of these two quantities reflect the actual amount captured at 0.1 bar and released at 1.0 bar, because both states 3′_i_ and 1 are at *p*_1_ = 0.1 bar while both states 1′ and 3 are at *p*_3_ = 1 bar. The important quantity is the difference in DIC between states 3 and 1. With help of TA and pH measurements, ΔDIC_flow,1→3_ is evaluated as ΔDIC_TA−pH,1→1′_ + ΔDIC_flow,1′__→3_ = −0.17 M; equivalently, but with opposite sign, ΔDIC_flow,3→1_ = ΔDIC_flow,3′__→1_ + ΔDIC_TA−pH, 3→3′__f_ = 0.17 M, i.e., 1.7 mmol CO_2_ in a 10 mL negolyte volume. Because sufficient gas-solution equilibrium is approached (Fig. 2f, g), ΔDIC_TA−eq_ may also be used in place of ΔDIC_TA−pH_ in such calculations, resulting in the same values of ΔDIC_flow,1→3_ and ΔDIC_flow,3→1_.

In this cycle, the deacidification work into the system, *w*_deacidification_, is 0.267 kJ and the acidification work, *w*_acidification_, is −0.119 kJ (Eq. 3). Dividing the cycle work, *w*_cycle_ (Eq. 2), by 1.7 mmol CO_2_ gives the molar cycle work of 87 kJ mol_CO2_^−1^. This value is already competitive with commercial amine scrubbing processes^4,6^, and it can be further decreased by using membranes with lower ohmic resistance or molecules with lower electron transfer overpotential^22^.

The productivity measures the rate of a CO_2_ separation process and may be evaluated by dividing ΔDIC_flow,3→1_, i.e., 0.17 M, by the sum of the absorption time and the desorption time. The CO_2_ absorption and desorption processes took 1.6 and 0.4 h, respectively, leading to a productivity of 0.085 M h^−1^ or 8.5 $\times$ 10^−4^ mol_CO2_ h^−1^. The productivity should vary monotonically with current density because the desorption process is mostly limited by the rate of TA consumption, which is proportional to the rate of electrochemical oxidation. The solution-gas contacting area is quite limited in this experiment: gas was simply bubbled through the solution at a low rate (11.8 mL/min). Engineered contactor structures have many orders of magnitude higher contact area. Because the sorbent in this process is aqueous KOH, the contactor that is used for the concentrated alkaline process^9^ might be adopted, for example, and a similar capture rate would be expected. Other factors such as solution concentration, gas flow rate, etc. also influence the productivity but analysis of such dependencies is beyond the scope of this study.

### Carbon capture cycles with *p*_1_ = 0.1–0.5 bar at 40 mA cm^−2^

**Fig. 3:**
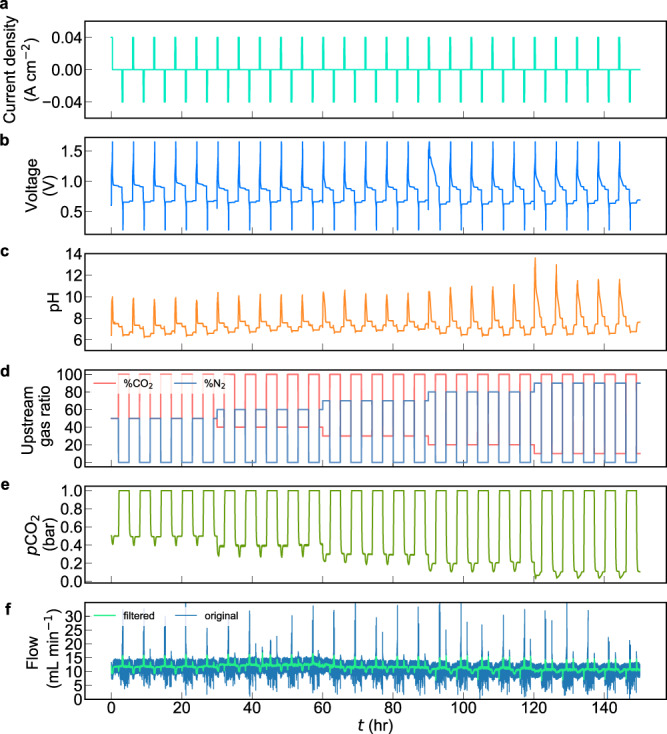
Twenty five CO_2_ concentrating cycles with 0.5, 0.4, 0.3, 0.2, and 0.1 bar inlet pCO2 and 1 bar exit pCO2 at 40 mA cm^−2^. Same cell and negolyte as in Fig. 2 were employed**. a** Current density. **b** Voltage. **c** pH of the negolyte. **d** N_2_ and CO_2_ percentage in the upstream source gas, controlled by mass flow controllers; total pressure 1.0 bar. **e** Downstream CO_2_ partial pressure. **f** Downstream total gas flow rate.

**Fig. 4:**
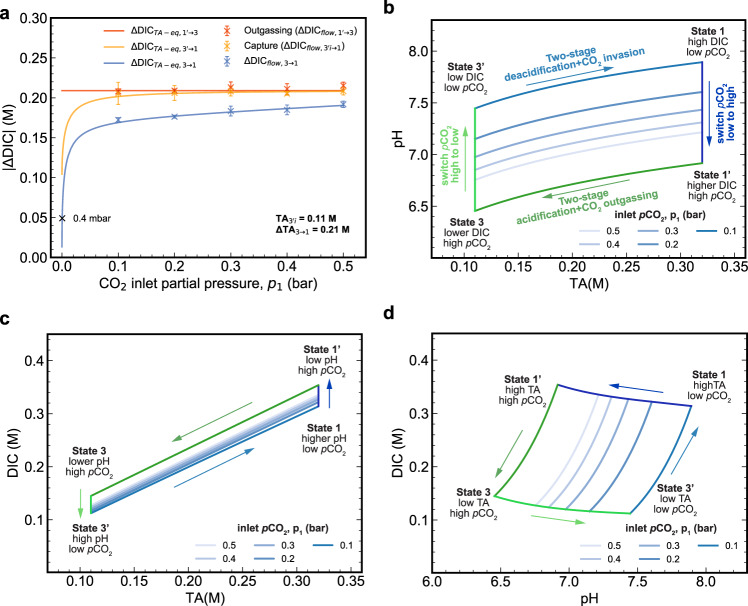
Summary of the experimental concentrating cycles with different inlet *p*_CO2_ in Fig. 3 and the TA (total alkalinity)/pH/DIC (dissolved inorganic carbon) relations of the ideal cycles with corresponding experimental conditions. **a** ΔDIC_flow_ extracted from Fig. 3e, f (colored “x” markers) and calculated ΔDIC_TA−eq_ given TA_3′i_ = 0.11 M and ΔTA_3→1_ = 0.21 M (lines). The black “x” marker refers to the result that ΔDIC for the ideal cycle equals 0.049 M when *p*CO_2_ = 0.4 mbar. The error bars refer to the standard deviation. **b** pH *vs*. TA in the ideal cycles, assuming TA_3′__i_ = 0.11 M, ΔTA_3→1_ = 0.21 M and gas-solution equilibrium. *p*_1_ in the legends represents *p*CO_2_ during the two-stage deacidification+CO_2_ invasion process. The arrows indicate the direction of the processes in the experiments. **c** DIC *vs*. TA in the ideal cycles. **d** DIC *vs*. pH in the ideal cycles.

In order to understand how the electrical work depends on the inlet *p*CO_2_, we performed five cycles each at *p*_1_ = 0.1 to 0.5 bar with *p*_3_ = 1.0 bar (Fig. 3). The same cell components and negolyte as in Fig. 2 were used, and the posolyte was replaced with a fresh solution for each inlet condition to avoid oxygen-induced long-term cell imbalance (Fig. 1c)^23^. Supplementary Figure 5b shows that the CO_2_ outgassing period is identical regardless of inlet *p*CO_2_ because the exit *p*CO_2_ is always 1 bar and the current density is always 40 mA cm^−2^. In contrast, the capture period increases as inlet *p*CO_2_ decreases (Supplementary Fig. 5a) because of the expected trend of reaction rate with decreasing reactant concentration. ΔDIC_flow,3→1_ values decrease as *p*_1_ decreases (Fig. 4a) because of greater ΔDIC during processes 1→1’ and 3→3’ (vertical arrows in Fig. 4c) at smaller *p*_1_. The measured values closely align with the theoretical ΔDIC_TA−eq,3→1_ vs. *p*_1_ curve (Fig. 4a).

This alignment permits us to estimate ΔDIC_fow,3→1_ for *p*_1_ = 0.4 mbar and *p*_3_ = 1 bar, i.e., similar to DAC conditions, by following the ΔDIC_TA−eq,3→1_ vs. *p*_1_ curve to obtain a value of 0.049 M. Note that the ΔDIC_TA−eq, 3→1_ vs. *p*_1_ curve shifts downward as TA_3’i_ increases (Supplementary Fig. 3b). This has relatively small impacts on ΔDIC_TA−eq,3→1_ with high *p*_1_, but it causes significant differences for small *p*_1_ values. For example, when *p*_1_ = 0.4 mbar, ΔDIC_TA−eq,3→1_ for TA_3′i_ = 0, 0.11 and 0.21 M is 0.097, 0.049 and 0.005 M, respectively (Supplementary Figs. 1–3). Because ΔDIC_3→1_ is in the denominator when CO_2_ molar cycle work is calculated, lowering ΔDIC_3→1_ increases the molar energy cost accordingly (Supplementary Fig. 2b, c). High TA_3’i_ should therefore be avoided, and a necessary step to achieve this goal is to limit the impact of oxidation of DSPZH_2_ by oxygen (Fig. 1c).

### Carbon capture cycles with *p*_1_ = 0.1–0.5 bar at 20–150 mA cm^−2^

**Fig. 5:**
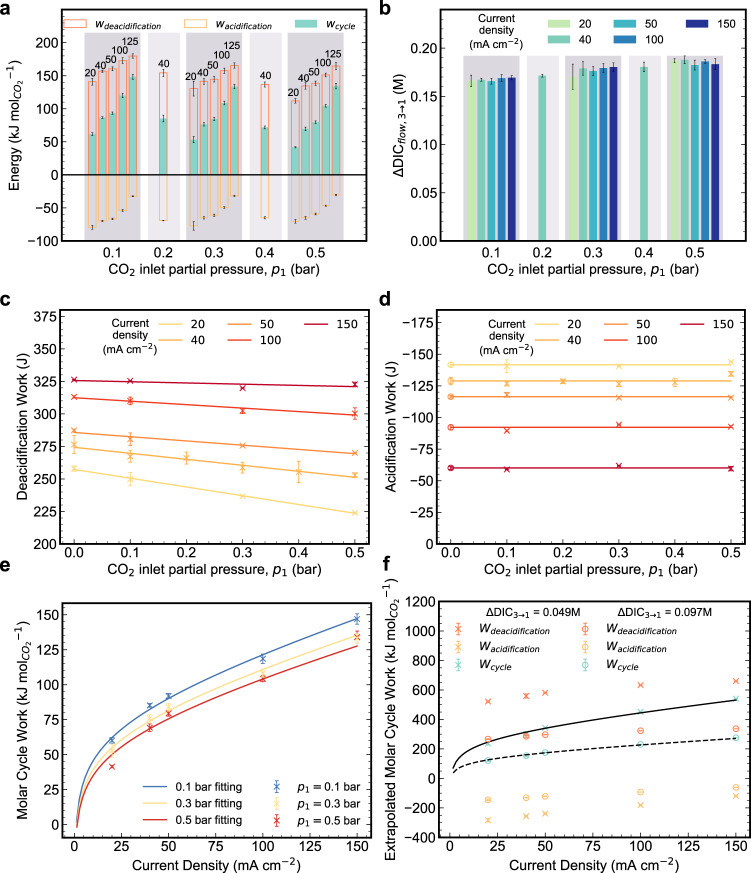
Summary of the experimental concentrating cycles performed under 20, 40, 50, 100 and 150 mA cm^−2^ current densities and *p*_1_ = 0.5, 0.4, 0.3, 0.2 and 0.1 bar. TA is total alkalinity and DIC is dissolved inorganic carbon. Electrolytes comprised 10 mL of 0.11 M DSPZ in 1 M KCl (negolyte) and 35 mL of 0.1 M K_4_Fe(CN)_6_ and 0.04 M K_3_Fe(CN)_6_ in 1 M KCl (posolyte). The error bars refer to standard deviation. **a** CO_2_ molar deacidification, acidification, and cycle work *vs. p*_1_ for current densities indicated above the bars, in mA cm^−2^. In both (**a**) and (**b**) the horizontal axis is categorical, and each shadowed region refers to a single *p*_1_ value. **b** ΔDIC_flow,3→1_ *vs p*_1_ for various current densities. **c** Deacidification work *vs. p*_1_ for various current densities. The “x” markers refer to measured data. The deacidification work of the cycles under pure N_2_ is used for *p*_1_ = 0.0 bar. **d** Acidification work vs. *p*_1_ for various current densities. The “x” markers refer to measured data. For each current density, the acidification work at *p*_1_ = 0.0 bar (“o” markers) is chosen to be the average value of the work obtained at other *p*_1_ values at the same current density. **e** CO_2_ molar deacidification, acidification and cycle work vs. current density for *p*_1_ = 0.1, 0.3 and 0.5 bar. The curves are fitted using a Tafel model. **f** Extrapolated CO_2_ molar deacidification, acidification and cycle work for *p*_1_ = 0.4 mbar. Extrapolation is performed using deacidification and acidification work at 0.0 bar *p*_1_ in (**c**) and (**d**), and divided by ΔDIC_TA–eq,3→1_ at *p*_1_ = 0.4 mbar obtained from Fig. 4a and Supplementary Fig. 2b. The solid line refers to a Tafel model fit of CO_2_ molar cycle work vs. current density assuming TA_3’i_ = 0.11 M (ΔDIC_3→1_ = 0.049 M) and the dashed line refers to the same fitting but assuming TA_3′i_ = 0.0 M (ΔDIC_3→1_ = 0.097 M).

The average CO_2_ molar cycle work under 40 mA cm^−2^ is compared with those obtained under 20–150 mA cm^−2^ (Fig. 5a and Supplementary Fig. 7). As current density increases at a fixed *p*_1_, the net cycle work increases as the required deacidification work increases and the acidification work returned decreases in magnitude; these trends are caused by increased ohmic, electron-transfer, and mass-transport overpotentials at higher current density^22^. It is noteworthy that we achieve 61.3 kJ mol_CO2_^−1^ cycle work for *p*_1_ = 0.1 bar and *p*_3_ = 1 bar using a current density of 20 mA cm^−2^, which is a competitive energy cost at a much higher current density compared to other electrochemical CO_2_ separation methods for flue gas capture^24,25^.

It is evident from Fig. 5b that ΔDIC_flow,3→1_ is independent of current density for a given value of *p*_1_. This occurs because varying current density changes only the rate of change in TA and not the value of ΔTA_3→1_, and sufficient reaction time was allowed to approach gas-solution equilibrium, whether the current density was low (Fig. 2f, g) or high (Supplementary Fig. 9). The consistent ΔTA_3→1_ across various current densities is supported by the consistent charge/discharge capacities (Supplementary Fig. 8). The slight variations in ΔTA and ΔDIC were caused by occasional foaming or negolyte droplets clinging to the wall of the reservoir, both of which cause small amounts of charge capacity to be instantaneously inaccessible from time to time. In contrast, increasing *p*CO_2_ at the inlet raises ΔDIC_flow,3→1_ (Fig. 5b), for the reason explained in the discussion of Fig. 4d. In addition to the cycle results presented in Fig. 5, five cycles with *p*_1_ = 0.05 bar and *p*_3_ = 1 bar and current density being 40 mA cm^−2^ were tested under faster negolyte pumping to enhance mass transport rates, yielding an average cycle work of 92.6 kJ mol_CO2_^−1^ (Supplementary Fig. 10).

### Estimate of molar cycle work at *p*_1_ = 0.4 mbar and *p*_3_ = 1 bar

Because of the limited sensitivity of our equipment, a direct measurement of CO_2_ molar cycle work at *p*_1_ = 400 ppm and *p*_3_ = 1 bar, i.e., similar to DAC conditions, is currently infeasible in our laboratory, but we can extrapolate the molar cycle work under these conditions using the results obtained from *p*_1_ = 0.1–0.5 bar. However, a simple linear regression of the molar cycle work from higher *p*_1_ values to *p*_1_ = 0.4 mbar does not guarantee accurate extrapolation because the deacidification work (Fig. 5c), i.e., part of the numerator in the calculation of molar cycle work (Eqs. (2)–(4) in the “Methods” section), scales linearly with *p*_1_, whereas ΔDIC_flow,3→1_, i.e., part of the denominator in the calculation of molar cycle work (Eq. (4) in the method section), scales non-linearly with *p*_1_ (Fig. 4a, d and Supplementary Fig. 3b). Therefore, we evaluate the molar cycle work at *p*_1_ = 0.4 mbar by separately calculating ΔDIC_flow,3→1_ and the cycle work (i.e., the sum of the experimental deacidification work (Fig. 5c) and the acidification work (Fig. 5d)). The deacidification work at *p*_1_ = 0.4 mbar is simply approximated by the deacidification work under a pure N_2_ atmosphere, i.e., 0.0 bar *p*CO_2_ (Fig. 5c); the reason that deacidification work decreases with increasing *p*_1_ is that increasing *p*_1_ lowers the average negolyte pH, which in turn decreases the cell voltage and thereby decreases the work (Eq. 3 in the “Methods” section). The acidification work is always the same regardless of *p*_1_ because *p*_3_ is always 1 bar and ΔTA_3→1_ is always the same (hence the flat lines in Fig. 5d); therefore, for *p*_1_ = 0.0 bar we use the average acidification work from higher *p*_1_ values. As for ΔDIC_flow,3→1_ at *p*_1_ = 0.4 mbar, we assume it is equal to the value of ΔDIC_TA–eq,3→1_ in the ideal cycle at the same pressure. We have shown that measured (ΔDIC_flow,3→1_) and ideal cycle (ΔDIC_TA–eq,3→1_) values of ΔDIC_3→1_ at *p*_1_ = 0.1–0.5 bar agree very well (Fig. 4a, blue curve). With TA_3’i_ = 0.11 M and ΔTA_3→1_ = 0.21 M, the ideal cycle value of ΔDIC_TA–eq,3→1_ at *p*_1_ = 0.4 mbar and *p*_3_ = 1 bar is 0.049 M (Fig. 4a, Supplementary Fig. 2b). The molar cycle work for various current densities, evaluated by dividing the sum of deacidification and acidification work at *p*_1_ = 0.0 bar by ΔDIC_TA–eq,3→1_ of 0.049 M, is shown in Fig. 5f. Figure 5f suggests that the molar cycle work at 20 mA cm^−2^ is 237.4 kJ mol_CO2_^−1^. This is on par with the concentrated KOH process^9^. However, if there is no initial alkalinity, i.e., TA_3’i_ = 0.0 M, and the same ΔTA_3→1_ = 0.21 M is kept, the cycle work may be cut in half to 121.0 kJ mol_CO2_^−1^; this occurs because of the nearly doubled ΔDIC_flow,3→1_ of 0.097 M despite similar cycle work (Supplementary Figs. 2 and 3). The non-linear molar cycle work trend was fitted with a Tafel model, which suggests 72.8 and 98.1 kJ mol_CO2_^−1^ at 5 and 10 mA cm^−2^, respectively (ESI, Non-Linear Fit of Molar Cycle Work With Tafel Model). In addition, due to its solubility of 0.7 M in aqueous solution^18^, DSPZ can induce a ΔTA_3→1_ or Δ[OH^−^] of 1.4 M and thereby potentially yield even lower molar cycle work (Supplementary Fig. 4)

### Comparison with existing technologies

Table 2 summarizes some of the emerging technologies for point source capture, DAC, and direct ocean capture (DOC), where CO_2_ is removed from seawater, allowing more CO_2_ uptake by the ocean. Many approaches to DAC have used aqueous alkaline solutions^2,9^ or solid amine-based adsorption methods^2,24^, which require thermal excursions to release captured CO_2_. One of the state-of-the-art DAC approaches relies on concentrated (2–5 Molar) alkaline solutions on a high-area contactor to absorb CO_2_ and transform it into aqueous K_2_CO_3_ and KHCO_3_. These are then converted into solid CaCO_3_ in a pellet reactor by mixing the aqueous carbonates with Ca(OH)_2_. Releasing the CO_2_ requires heating the CaCO_3_ to 900 °C in an oxygen-fired calciner, which costs 264–296 kJ/molCO_2_^2,9^. Another, less mature, aqueous approach uses amino acids for the carbon capture step and undergoes a subsequent sorbent regeneration cycle employing solid bis-iminoguanidine carbonate precipitation and CO_2_ release through heating to >100 °C; this cycle requires 152–422 kJ/molCO_2_, depending on the type of guanidine, because a significant portion of the energy contributes to removing the undesirable hydrate from guanidine carbonate crystal^10,16^. Solid sorbent DAC, mostly based on solid amine absorption and release through thermal and/or pressure swing, allows reduced heating requirements (~100 °C), but amine decomposition may lead to high operational and end-of-life costs^2,26–28^.

**Table 2:** Comparison of this work and emerging technologies for DAC, DOC, and point source capture. CO_2_ separation work with “th” subscript denotes thermal energy inputs, whereas “e” subscript denotes electrical work input.

Method	Purpose	CO_2_ separation work inputs (kJ mol_CO2_^−1^)	Current density (mA cm^−2^)
Alkaline solvent^2,9^	DAC	264–396_th_^a^	N/A
Solid amine sorbents^2^	DAC	150–211_th_^b^	N/A
Amino acid solvents and solid bis-iminoguanidines^10^	DAC	152–422_th_^c^	N/A
Fuel cell concentrator^17^	DAC	350_e_^d^	0.5
Electrochemical alkaline sorbent regeneration^31^	DAC	374_e_^f^	0.5
Processing seawater within a BPMED reactor^13^	DOC	155_e_^g^	3.3
Titrating seawater with BPMED acid/base^12^	DOC	394_e_^h^	100
Traditional amine ab-/desorption^4^	Point source capture	132–150_th_	N/A
Amine ad-/desorption with advanced flash stripper^32^	Point source capture	92_th_^i^	N/A
Shell Cansolv^6^	Point source capture	103_th_	N/A
Petra Nova^33^	Point source capture	89_th_^j^	N/A
Quinone Direct binding^7^	Point source capture	56_e_^k^	0.5
EMAR^8^	Point source capture	30–113_e_^l^	2.7–11.8
*This work*	0.1 bar capture	61–145_e_	20–150
	0.4 mbar capture	121–237_e_	20 (extrapolated)

^a^Work input excludes electrical work required to operate air–liquid contactor, pellet reactor, and auxiliary equipment.

^b^Desorption energy for mid-range scenario; work input excludes electrical work required to operate air contactor fans and desorption vacuum pump.

^c^Energy required for bis(iminoguanidine) regeneration.

^d^Hydrogen gas is the energy source; Energy required to operate water cooling system is excluded.

^f^The process starts with a bicarbonate/carbonate solution, mimicking a solution saturated with DIC under 0.4 mbar inlet *p*CO_2_. The value is the required work for alkaline sorbent solution regeneration.

^g^Work input excludes costs for ocean water intake, pre-treatment, and pumping.

^h^Energy consumption for the best-case acid process; work input excludes electrical work required to operate pumps and chiller.

^i^The inlet gas source contains 11.3% CO_2_, and the exit is 99% CO_2_.

^j^The inlet gas source contains 11% CO_2_, and the exit is 97% CO_2_. This energy is calculated using the electrical power cost, excluding 50% used for compression, plus the steam cost associated with the CCS plant. The captured CO_2_ was offset by the CO_2_ emission in the CCS plant.

^k^The inlet gas source was simulated flue gas with 15% CO_2_ and 3% O_2_ in N_2_, and exit partial pressure was ~0 bar. Note that the energy cost was calculated based on the amount of CO_2_ absorbed, yet it is not clear that all absorbed CO_2_ was released.

^l^Energy and current density values adopted from Fig. 8a of ref. ^8^. Simulated flue gas is 15% CO_2_ in N_2_.

Electrochemical carbon capture methods may offer solutions to overcome the high sorbent regeneration energy penalty and sorbent decomposition issues. Electrochemically mediated point source carbon capture methods^7,24,29^, at low current densities, have exhibited lower energetic costs than amine-scrubbing methods. In addition, CO_2_ removal from ocean water, which restores the CO_2_ capture capability of oceans, via electrochemical methods such as bipolar membrane electrodialysis (BPMED), have shown promisingly low energetic cost^12,13^. However, the demonstrated works exhibited either low current density (slow kinetics) or low voltage efficiency. In addition, the high water-handling requirement of direct ocean capture adds significantly to the energetic cost.

The performance of our pH-swing flow cell, demonstrated for capture at 0.1 bar and projected for 0.4 mbar appears competitive compared with existing technologies, not only in terms of energetic cost with cheap electricity from renewable sources, but also because of much larger applicable current density (Table 2)^30^. Additionally, the all-liquid configuration obviates the need for the precipitation and heating of solid carbonates. Furthermore, the compatibility with an aqueous electrolyte of non-volatile, non-corrosive and potentially low-cost organic molecules implies that a carbon capture technology based on this concept has the potential for wide scale practical implementation.

### Electrochemical rebalancing

**Fig. 6:**
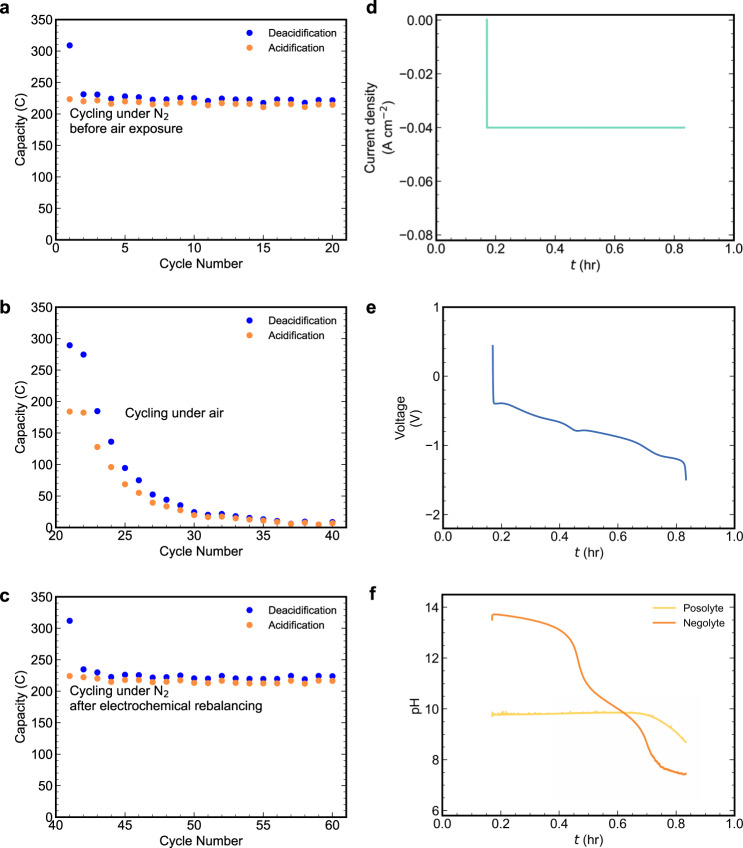
The capacity fade caused by O_2_ on Fe(CN)6|DSPZ cell cycling (a–c) and its mitigation by the electrochemical rebalancing method (d–f). **a** Charge capacity vs. cycle number of the cell under pure N_2_ atmosphere. The first cycle has much higher deacidification capacity due to residual oxygen. **b** Charge capacity vs. cycle number of the same cell from (**a**) under air. Capacity fades quickly because of the depletion of K_4_Fe(CN)_6_ in the posolyte. **c** Charge capacity *vs*. cycle number of the cell from (**b**) under pure N_2_ atmosphere, after the electrochemical rebalancing step. The first cycle has much higher deacidification capacity due to residual oxygen. **d** Current density, (**e**) voltage, (**f**) posolyte and negolyte pH during the electrochemical rebalancing step.

It is clearly difficult to avoid O_2_ in either DAC or flue gas capture because the source gas contains 20% and 3−5% O_2_, respectively. In the short term, the oxidation of DSPZH_2_ by O_2_ incurs an instantaneous loss in Coulombic efficiency. In the long term the cell will go out of balance, accumulating oxidized species in both electrolytes and TA_3’i_, KOH, and DIC_3’i_ in the negolyte (Fig. 1c)^23^. As a result, ΔDIC_3→1_ will shrink without a concomitant decrease in cycle work (Supplementary Fig. 2b), leading to an increase in CO_2_ molar cycle work (Supplementary Fig. 2c). Eventually, the cell will no longer operate because both electrolytes are completely oxidized. As shown in Fig. 6a, b, as soon as the headspace was opened to air the Coulombic efficiency decreased to ~65%, and by the 20th subsequent cycle the cell lost all capacity due to depletion of reduced species, i.e., [K^+^]_4_[Fe^II^(CN)_6_]^4−^, in the posolyte side. The negolyte pH also increased from near neutral to almost 14 during air exposure (Supplementary Fig. 14b). Development of oxygen-insensitive molecules may alleviate the problem caused by oxygen, but even if a tiny amount of Coulombic efficiency loss, e.g., 0.1%, took place every cycle, the effect is cumulative and will eventually lead to an out-of-balance cell problem (Fig. 1c).

Here we demonstrate the efficacy of the electrochemical rebalancing method. The method successfully recovers the pH of the negolyte and the capacity of the cell, which is thrown out-of-balance by O_2_-induced side reactions. The electrochemical rebalancing process comprises the cathodic reaction [K^+^]_3_[Fe^III^(CN)_6_]^3−^+ *e*^−^ → [K^+^]_4_[Fe^II^(CN)_6_]^4−^ in the posolyte and the anodic oxygen evolution reaction, OH^−^ → 2*e*^−^ + ½O_2_, in the negolyte. Figure 6d–f shows the cell behavior when the electrochemical rebalancing process is applied to the completely out-of-balance cell (Fig. 6b). The process starts when a constant current of −40 mA cm^−2^ is applied (Fig. 6d). The voltage immediately drops from 0.2 V to negative values because both the cathodic and anodic half reactions are at ~0.4 V vs. SHE at pH 14, and there is high activation overpotential for the oxygen evolution reaction (Fig. 6e). As the rebalancing process progresses, the pH of the negolyte side decreases (Fig. 6f), causing the anodic half reaction to shift to higher potential, thereby further decreasing the cell potential (Fig. 6e). The sharp drop in voltage to a plateau near 0.8 h indicates the completion of the electrochemical rebalancing process. Figure 6c shows the post-rebalancing cell capacity, which is almost identical to that prior to air exposure (Fig. 6a), indicating that essentially all lost capacity due to imbalance has been restored. The capacity accounting for all the electrons passed in the electrochemical rebalancing step is 476.8 C, which is within 1% of the theoretical capacity (473 C) of the posolyte side, suggesting a complete recovery of the K_4_Fe(CN)_6_ and minimal side reactions other than oxygen evolution. The posolyte pH did not change much during the process because the cathodic half-reaction is not proton-coupled (Fig. 6f). The neutral pH of the negolyte at the end of the process indicates that virtually all of the accumulated hydroxide has been removed (Fig. 6f). The undiminished capacity also suggests that this method is not detrimental to DSPZ. Supplementary Figure 14 shows that the electrolytes, after electrochemical rebalancing, have the same carbon-capture capability as the original electrolytes, and the NMR spectra in Supplementary Fig. 15 suggest no new species was generated during the process. Hence, the electrochemical rebalancing process is a very effective method to remove the adverse effect of oxygen in DSPZ-based carbon capture flow cells. This method has potentially broad application beyond DSPZ and carbon capture, e.g., mitigating the oxygen effect in flow batteries with air or pH-sensitive electrolytes (ESI, More on Electrochemical Rebalancing)^23,34–41^. The overall energy cost is 378 J, which is approximately 1.4 times of the cost of one deacidification half cycle at 40 mA cm^−2^ (Fig. 5c). This will be a significant cost if the electrochemical rebalancing is applied every few cycles, which may be necessary for a DSPZ-based system for DAC, but if the negolyte molecule is much less air sensitive or the source gas has lower oxygen content, requiring electrochemical rebalancing less frequently than once every few tens of carbon capture/release cycles, the cost will be negligible. The development of oxygen-insensitive molecules for this purpose is the subject of active research.

In this work, we have performed a series of CO_2_ concentrating cycles using a DSPZ-based flow cell with electrochemically induced pH swings, and the cycle work under different inlet partial pressures and current densities was analyzed and compared. We demonstrated a 61.3 kJ mol_CO2_^−1^ cycle work for CO_2_ separation for capture at *p*_1_ = 0.1 bar and release at *p*_3_ = 1 bar, at a current density of 20 mA cm^−2^. If TA_3′i_ is carefully maintained at a low level, the extrapolated separation work for *p*_1_ = 0.4 mbar and *p*_3_ = 1 bar is 121 kJ mol_CO2_^−1^ at 20 mA cm^−2^ and this figure can be further lowered if a higher concentration of DSPZ or another PCET-active molecule is used. Our Tafel model suggests that molar cycle work at *p*_1_ = 0.4 mbar may be even lower than 100 kJ mol_CO2_^−1^ at smaller current densities_._ Recognizing the inevitable O_2_-induced imbalance and capacity fade in both point source capture and DAC, we report an electrochemical rebalancing method that recovers the initially healthy cell composition. This method can serve as a convenient tool for mitigating oxygen-related problems in many electrochemical applications. We anticipate that the low energetic cost of the pH swing cycles and the effectiveness of the oxygen mitigation method demonstrated here will accelerate the techno-economic competitiveness of electrochemically-driven carbon capture systems.

## Methods

### Materials and characterization

All chemicals were purchased from Sigma-Aldrich or Acros Organics and were used as received. The synthetic method for DSPZ is adapted from previous work^18^. In this work, sodium hydride was used to deprotonate the reaction intermediate phenazine-2,3-diol (DHPZ) instead of sodium methoxide (Supplementary Fig. 17).

### Flow cell experiments

Flow cell experiments were constructed with cell hardware from Fuel Cell Tech. (Albuquerque, NM), assembled into a zero-gap flow cell configuration, similar to a previous report^18^. Pyrosealed POCO graphite flow plates with serpentine flow patterns were used for both electrodes. Each electrode comprised a 5 cm^2^ geometric surface area covered by a stack of four sheets of Sigracet SGL 39AA porous carbon paper pre-baked in air for 24 h at 400 °C. The outer portion of the space between the electrodes was gasketed by Viton sheets with the area over the electrodes cut out. Torque applied during cell assembly was 80 lb-in on each of eight 1/4-28 bolts. The membrane used is a Fumasep E620(K) CEM. Cell electrolytes comprised 10 mL 0.11 M DSPZ in 1 M KCl (negolyte, capacity limiting, theoretical capacity 212 C) and 35 mL 0.1 M K_4_Fe(CN)_6_ and 0.04 M K_3_Fe(CN)_6_ in 1 M KCl (posolyte, non-capacity limiting, theoretical capacity 473 C). For every new CO_2_ capture cycle condition (changing current density or inlet *p*CO_2_), the posolyte was replaced with a fresh solution and the negolyte was acidified by adding drops of 1 M HCl to remove the accumulating effect of oxygen side reactions. 10 μL of antifoam B emulsion purchased from Sigma-Aldrich was added into the negolyte solution before cell cycling to suppress foam formation. Posolytes were fed into the cell through fluorinated ethylene propylene (FEP) tubing at a rate of 100 mL min^−1^ controlled by a Cole-Parmer 6 Masterflex L/S peristaltic pump, and the negolytes were circulated at the same rate controlled by a Cole-Parmer Masterflex digital benchtop gear pump system. Both posolyte and negolyte upstream gas was controlled by Sierra Smart Trak 50 Mass Flow Controllers. The flowmeter used in the downstream of negolyte headspace was a Servoflo FS4001-100-V-A. The CO_2_ sensor was an ExplorIR-W 100% CO_2_ sensor purchased from co2meter.com. A Mettler Toledo pH electrode LE422 was used to monitor electrolyte pH. As shown in Fig. 1b, a drierite drying tube (Cole Parmer) was placed in between the sensors and the negolyte chamber to reduce the humidity level of the gas.

Glassy carbon (BASi MF-2012, 3.0 mm diameter) was used as the working electrode for all three-electrode CV tests. A Ag/AgCl reference electrode (BASi MF-2052, pre-soaked in 3 m NaCl solution), and a graphite counter electrode were used for CV tests. CV tests and cell cycling were performed using a Gamry Reference 3000 potentiostat. All cycles were galvanostatic until the 1.65 and 0.2 V voltage cutoff for deacidification and acidification, respectively, were reached, and then went through a potentiostatic process until the current reached 10 mA cm^−2^. In the CO_2_ cycles with *p*_1_ = 0.1, 0.2, 0.3, 0.4, and 0.5 bar, the MFCs set the initial negolyte headspace atmosphere to be *p*_1_, which was then switched back and forth between *p*_1_ and *p*_3_ every three hours. In the cycles with *p*_1_ = 0.05 bar, the switching period was 5 h.

### Calculation of absorbed or released CO_2_ amount

Because the deviation from baseline in Fig. 2g is solely caused by CO_2_ absorption, the amount of CO_2_ captured is calculated by integrating over the difference between the recorded flow rate and the baseline in between 0.2 and 1.8 h, i.e.,

1 $${Q}_{{{{{{\rm{C{O}}}}}}}_{2}}=\mathop{\sum }\limits_{n={t}_{i}}^{{t}_{f}}({\dot{V}}^{{{{{{\rm{base}}}}}}}-{\dot{V}}^{n})\Delta t$$

where ${Q}_{{{{{{\rm{C{O}}}}}}}_{2}}$ is the volume of CO_2_, *t*_*i*_ is the start time, *t*_*f*_ is the final time, *V̇*^*n*^ is the instantaneous volumetric flow rate at *n*th data recording time *t*_*n*_, *V̇*^base^ is the baseline flow rate of 11.6 mL min^−1^, and Δ*t* is the time difference between successive measurements.

### Calculation of deacidification, acidification, and cycle work

The net cycle work is calculated by combining the work required for deacidification in process 3′_i_→1 and the work returned by acidification in process 1′→3, i.e.,

2 $${w}_{{{{{{\rm{cycle}}}}}}}={w}_{{{{{{\rm{deacidification}}}}}}}+{w}_{{{{{{\rm{acidification}}}}}}}$$

The work in a process is calculated by summing over the product of voltage (Fig. 2a) and current (Fig. 2b), i.e.,

3 $${w}_{{{{{{\rm{deacidification}}}}}}/{{{{{\rm{acidification}}}}}}}=\mathop{\sum }\limits_{n={t}_{i}}^{{t}_{f}}{V}^{n}{j}^{n}A\Delta t$$

where *V*^*n*^ is the cell voltage at the *n*th data recording time *t*^*n*^, *j*^*n*^ is the current density at *t*^*n*^ and *A* is the active geometric area of 5 cm^2^.

The molar cycle work $\bar{w}$ is calculated by dividing *w*_cycle_ by −ΔDIC _flow,1→3_ or ΔDIC_flow,3→1_:

4 $$\bar{w}=\frac{{w}_{{{{{{\rm{cycle}}}}}}}}{\varDelta {{{{{\rm{DIC}}}}}}_{{{{{{\rm{flow}}}}}},3\to 1}}$$

where ΔDIC_flow,1→3_ = ΔDIC_TA−pH,1→1′_ + ΔDIC_flow,1′__→3_ and ΔDIC_flow,3→1_ = ΔDIC_flow,3′__→1_ + ΔDIC_TA−pH, 3→3′__f_. For high current densities (100 and 150 mA cm^−2^), we use ΔDIC_TA−eq,1→1′_ and ΔDIC_TA−eq, 3→3′__f_ instead of ΔDIC_TA−pH,1→1′_ and ΔDIC_TA−pH, 3→3′__f_, respectively, because of an artifact in the pH measurement at high current density, tentatively attributed to crosstalk between potentionstat lines. We explain in the main text that ΔDIC_TA−pH_ and ΔDIC_TA−eq_ are interchangeable when the pH measurement is valid.

## Supplementary information

Supplementary Information

## Data Availability

Source data are provided with this paper and available at https://github.com/martin94jjj/2022_nat_comm_CO2_data. Source data are provided with this paper.

## Source data

Source Data

## Code availability

The code that supports the plots and discussion of this study is available from the corresponding author upon reasonable request.
