# Supplementary material for: Low energy carbon capture via electrochemically induced pH swing with electrochemical rebalancing
Source: Nat Commun. 2022 Apr 19;13:2140. doi: 10.1038/s41467-022-29791-7 (PMC9018824; doi:10.1038/s41467-022-29791-7)
Supplement: Supplementary file 1 — Supplementary Information [file 41467_2022_29791_MOESM1_ESM.pdf]

Electronic Supplementary Information

for

Low Energy Carbon Capture via Electrochemically Induced pH Swing with Electrochemical Rebalancing

Shijian Jin[1], Min Wu[1], Yan Jing[2], Roy Gordon[2] and Michael J. Aziz[1*]

[1] John A. Paulson School of Engineering and Applied Sciences, Harvard University, Cambridge, Massachusetts, 02138, United States

[2]Department of Chemistry and Chemical Biology, Harvard University, Cambridge, Massachusetts 02138, United States

*maziz@harvard.edu

Table of Additional Sections

1 CO_2 Molar Ideal Cycle Work 5

2 Cycle Data.. 10

3 Non-Linear Fit of Molar Cycles Work With Tafel Model 16

4 More on Electrochemical Rebalancing.. 19

5 Synthesis 23

Table of Additional Figures

Supplementary Figure 1| Ideal cycles constructed using p_1 = 0.4 mbar and p_3 = 1 bar, $\Delta TA_{3\rightarrow1}$ = 0.21 M and varying $TA_{3'}$. a-c, $TA_{3'}$ = 0.0 M; d-f, $TA_{3'}$ = 0.11 M and g-i, $TA_{3'}$ = 0.21 M; 7

Supplementary Figure 2| Dependence of (a) $w_{cycle,ideal}$, (b) $\Delta DIC_{TA-pH,3\rightarrow1}$ (c) and wideal on $TA_{3'}$ in the ideal cycles with p_1 = 0.4 mbar, and p_3 = 1 bar, $\Delta TA_{3\rightarrow1}$= 0.21 M. 8

Supplementary Figure 3| Dependence of (a) $w_{cycle,ideal}$, (b) $\Delta DIC_{TA-eq,3\rightarrow1}$ and (c) wideal on p_1 in the ideal cycles with $\Delta TA_{3\rightarrow1}$ = 0.21 M and $TA_{3'}$ = 0.0, 0.11 and 0.21 M. The 'x' marks indicate p_1 = 0.4 mbar. 8

Supplementary Figure 4| Dependence of (a) $w_{cycle,ideal}$, (b) $\Delta DIC_{TA-eq,3\rightarrow1}$ and (c) and wideal on $\Delta TA_{3\rightarrow1}$ in the ideal cycles with p_1 = 0.4 mbar, p_3 = 1 bar and $TA_{3'}$ = 0.0 M. 8

Supplementary Figure 5| Duration of the CO_2 capture and release processes in the cycles with 40 mA cm^{-2} current density. a, Downstream pCO_2 of one capture half cycle for each of the inlet pCO_2 conditions. b, Filtered total gas flow rate of one outgassing half cycle for each of the inlet pCO_2 conditions. All the processes look identical because of the same exit condition, so an arbitrary offset is added to differentiate the curves. 10

Supplementary Figure 6| Capture and outgassing durations extracted from Supplementary Figure 5 a and b. 10

Supplementary Figure 7| Eighty-five CO_2 concentrating cycles with varying inlet pCO_2 and current densities. These are the raw data for Fig. 5. Same cell was used as in Fig 2. Liquid pumping rate is 50 mL min^{-1} for all the cycles. Note that, the pH measurements for high current densities are inaccurate, as the pH should never be able to reach pH > 14 for 0.11 M DSPZ. a, Current density. b, Voltage. c, N_2 and CO_2 percentage in the upstream source gas, controlled by mass flow controllers. d, CO_2 partial pressure. e, Total gas flow rate. 11

Supplementary Figure 8| Charge (a) and discharge (b) capacities of each experimental condition. In both a and b the horizontal axis is categorical, each shadowed region refers to a single p_1 value. 12

Supplementary Figure 9| One CO_2 concentrating cycle from Supplementary Figure 7 with 0.1 bar inlet pCO_2 and 1 bar exit pCO_2 at 150 mA cm^{-2}. Note that, the pH measurements are not shown because of an inexplicable artifact only present at high current, so it is invalid to extract DIC_{TA-pH} for this condition. However, DIC_{TA-eq} can be extracted because sufficient gas-solution is reached, as demonstrated by fact that the *p*CO2 and flow curves return to their baselines after CO_2 invasion and outgassing. a, Voltage profile. b, Current density. c, N_2 and CO_2 percentage in the upstream source gas, controlled by MFC. d, CO_2 partial pressure. e, Total gas flow rate. 13

Supplementary Figure 10| Five CO_2 concentrating cycles with 0.05 bar inlet pCO_2 and 1 bar exit pCO_2 at 40 mA cm^{-2}. Same cell was used as in Fig. 2. Fresh negolyte and posolyte were used. The liquid pumping rate is 150 mL min^{-1}, which is 50% faster than for capture at higher inlet pressure. a, Voltage profile. b, Current density. c, pH of the negolyte. d, N_2 and CO_2 percentage in the upstream source gas, controlled by mass flow controllers. e, CO_2 partial pressure. f, Total gas flow rate. 14

Supplementary Figure 11| Post-electrochemical rebalancing CO_2 capture with 0.05 bar inlet pCO_2 and 1 bar exit pCO_2 at 40 mA cm^{-2}. Same cell was used as in Fig.2. Same posolyte and negolyte as in Supplementary Figure 10 were used. The liquid pumping rate is 150 mL min^{-1}. a, Voltage profile. b, Current density. c, pH of the negolyte. d, N_2 and CO_2 percentage in the upstream source gas, controlled by mass flow controllers. e, CO_2 partial pressure. f, Total gas flow rate. The system has the same carbon capture/release capability after the post-electrochemical rebalancing. 15

Supplementary Figure 12| The fitted curves η_{et} at p_1 = 0.1, 0.3 and 0.5 bar using the Tafel model. (a) η_{et} only and (b) the sum of η_{et} and η_{ohmic}. 18

Supplementary Figure 13| The fitted curves η_{et} at p_1 = 0.4 mbar using the Tafel model. $\Delta DIC_{3\to1}$ = 0.049 M when TA3'i = 0.11 M and $\Delta DIC_{3\to1}$ = 0.097 M when TA3'i = 0.0 M. (a) η_{et} only and (b) the sum of η_{et} and η_{ohmic}. 19

Supplementary Figure 14| pH of the negolyte during cycles before air exposure (a), under air (b) and after electrochemical rebalancing (c), respectively. pH drifts up because of oxygen presence. 19

Supplementary Figure 15| NMR spectra of aromatic region of (top) uncycled DSPZ, (middle) DSPZ after cycling under air, and (bottom) DSPZ after electrochemical rebalancing. No new peaks in the aromatic region were observed. The slight peak shifts were caused by concentration and pH differences. 20

Supplementary Figure 16| Cyclic voltammetry of DSPZ and 1 M KOH background. No additional peak was observed for DSPZ during the oxidative scan, indicating absence of side reactions.... 20

Supplementary Table 1| Table of acronyms

Acronyms	Explanation
BPMED	bipolar membrane electrodialysis
CEM	cation exchange membrane
DAC	direct air capture
DIC	dissolved inorganic carbon
DIC_x	concentration of dissolved inorganic carbon in state “x”
$\Delta DIC_{flow,x\rightarrow y}$	change in DIC between states “x” and “y” ($DIC_y - DIC_x$), measured by flow meter and CO_2 sensor
$\Delta DIC_{TA-pH,x\rightarrow y}$	change in DIC between states “x” and “y” ($DIC_y - DIC_x$), measured by known total alkalinity and measured pH
$\Delta DIC_{TA-eq,x\rightarrow y}$	change in DIC between states “x” and “y” ($DIC_y - DIC_x$), measured by by known total alkalinity and assuming gas-solution equilibrium
DOC	direct ocean capture
DSPZ	sodium 3,3’-(phenazine-2,3-diylbis(oxy))bis(propane-1-sulfonate)
$DSPZH_2$	reduced DSPZ
EMAR	electrochemically mediated amine regeneration
$K_3Fe(CN)_6$	potassium ferricyanide (oxidized form of $Fe(CN)_6$)
$K_4Fe(CN)_6$	potassium ferrocyanide (reduced form of $Fe(CN)_6$)
MFC	mass flow controller
p_1	CO_2 partial pressure in bar during CO_2 capture (inlet)
p_3	CO_2 partial pressure in bar during CO_2 outgassing (exit)
PCET	proton-coupled electron transfer
pH_{mea}	pH measured by pH probe
pH_{TA-eq}	pH calculated using known total alkalinity and assuming gas-solution equilibrium
TA	total alkalinity
TA_x	concentration of total alkalinity in state “x”
$\Delta TA_{x\rightarrow y}$	change in TA between states “x” and “y” ($TA_y - TA_x$), measured by counting charges during deacidification or acidification, which is equivalent to twice the concentration of DSPZ.

1 **CO_2 Molar Ideal Cycle Work**

For a system with given $TA_{3'i}$ and $\Delta TA_{3\to1}$, i.e. DSPZ concentration, the ideal cycle work is defined as the work input for driving the system through electrochemical deacidification at p_1 and a subsequent electrochemical acidification at p_3, at an infinitesimal current. In the ideal cycle, gas-solution equilibrium is assumed at every point and because TA is known, pH_{TA-eq} and DIC_{TA-eq} at every point can be calculated. The CO_2 molar ideal cycle work, which we denote as $\bar{w}_{ideal}$, is obtained from dividing the ideal cycle work by expected $\Delta DIC_{3\to1}$, i.e. $\Delta DIC_{TA-eq,3\to1}$.

This section explains how $\bar{w}_{ideal}$ is calculated in detail. Both of the ideal cycle work and $\Delta DIC_{TA-eq,3\to1}$ are governed by these parameters: initial TA ($TA_{3'i}$ or simply $TA_{3'}$ because $TA_{3'i}$ and $TA_{3'f}$ will be the same in an ideal cycle), $\Delta TA_{3\to1}$ and pCO_2 at p_1, and the following equations:

$$\mathrm{DIC}=[\mathrm{CO_2(aq)}]+[\mathrm{HCO_3^-}]+[\mathrm{CO_3^{2-}}]; \quad \text{(S1)}$$

$$\mathrm{K_1}=\frac{[\mathrm{HCO_3^-}][\mathrm{H^+}]}{[\mathrm{CO_2(aq)}]}; \quad \text{(S2)}$$

$$\mathrm{K_2}=\frac{[\mathrm{CO_3^{2-}}][\mathrm{H^+}]}{[\mathrm{HCO_3^-}]}; \quad \text{(S3)}$$

$$\mathrm{TA}\equiv[\mathrm{OH^-}]+[\mathrm{HCO_3^-}]+2[\mathrm{CO_3^{2-}}]-[\mathrm{H^+}]; \quad \text{(S4)}$$

$$[\mathrm{S^+}]-[\mathrm{S^-}]=\mathrm{TA}; \quad \text{(S5)}$$

$$[\mathrm{H^+}][\mathrm{OH^-}]=10^{-14}, \quad \text{(S6)}$$

where the K_1 and K_2 used here are 1.1×10^{-6} M and 4.1×10^{-10} M,[1] resulting in the first and second pKa for carbonic acid being 6.0 and 9.4, respectively. Eq. S4 is the definition of TA of the solution under consideration and eq. S5 arises from the charge neutrality constraint in solution (S^+ and S^- correspond to the cationic and anionic species of the electrolyte salt). During deacidification, $[S^+]$ increases in the negolyte reservoir, so TA increases as well (eq. S5), which means an increase of hydroxide concentration or $[HCO_3^-]$ or $[CO_3^{2-}]$ given nonzero pCO_2 (eq. S4). The reverse happens during acidification. The expressions for the concentration of each constituent of DIC can be derived by rearranging the above equations:

$$[\mathrm{CO_2(aq)}]=\frac{\mathrm{DIC}}{1+\frac{\mathrm{K_1}}{[\mathrm{H^+}]}+\frac{\mathrm{K_1K_2}}{[\mathrm{H^+}]^2}}; \quad \text{(S7)}$$

$$[\mathrm{HCO_3^-}]=\frac{\mathrm{DIC}}{1+\frac{[\mathrm{H^+}]}{\mathrm{K_1}}+\frac{\mathrm{K_2}}{[\mathrm{H^+}]}}; \quad \text{(S8)}$$

$$[CO_3^{2-}] = \frac{DIC}{1+\frac{[H^+]}{K_2}+\frac{[H^+]^2}{K_1K_2}};\qquad (S9)$$

$TA_{3'}$ is calculated using eq. S7, measured pH and assumed gas-solution equilibrium, i.e.

$$[CO_2(aq)] = 0.035 \times pCO_2. \qquad (S10)$$

where 0.035 comes from Henry's Law constant of 35 mM bar^{-1} at room temperature and the units of [CO_2(aq)] and pCO_2 are Molar and bars, respectively. For example, in **Table 1**, pH_{meas} at state $3'_i$ was 7.4, and pCO_2 was 0.1 bar, so DIC can be derived from eq. S7 and S10, and subsequently [HCO_3^-] and [CO_3^{2-}] from eq. S8 and S9, respectively. $TA_{3'i}$ is then obvious from eq. S4. Because $\Delta TA_{3'i\rightarrow 1}$, which is determined by the concentration of DSPZ, is equal to $\Delta TA_{3\rightarrow 1}$, and $-\Delta TA_{1'\rightarrow 3}$ (or $-\Delta TA_{1\rightarrow 3}$) in the ideal cycle, TA at states 1, 1' and 3 can be derived from $TA_{3'i}$ and ΔTA values. Because TA and pCO_2 is known for each state, pH_{TA-eq} and DIC_{TA-eq} can be calculated. Then $\Delta DIC_{TA-eq,3\rightarrow 1}$ is simply $DIC_{TA-eq,1}$ minus $DIC_{TA-eq,3}$. In fact, we can calculate TA, pH_{TA-eq} and DIC_{TA-eq} of every point in between the states as well, and hence construct the ideal cycles. Because of the $2H^+/2e^-$ redox processes of DSPZ,[2] its reduction potential, and overall cell potential decreases 59 mV for every unit of increase in pH. This allows us to calculate the ideal cycle work using the following equation:

$$w_{cycle,ideal} = \sum_{n=1} 0.059 \times (pH_{deacidification}(TA(n)) - pH_{acidification}(TA(n)) \times \Delta TA \times F \qquad (S11)$$

,where n is the index, TA increases by ΔTA Molar when n increases by 1, pH is a function of TA and the process, 0.059 (V/pH) is the conversion factor between pH and cell voltage, F is the Faraday constant (96485 C mol_{e-}^{-1}) and the unit of $w_{cycle,ideal}$ is J L^{-1}. Then $\bar{w}_{ideal}$ follows naturally by dividing $w_{cycle,ideal}$ by $\Delta DIC_{3\rightarrow 1}$.

$$\bar{w}_{ideal} = \frac{w_{cycle,ideal}}{\Delta DIC_{TA-eq,3\rightarrow 1}} \qquad (S12)$$

As mentioned in the main text, ΔDIC values vary as p_1, $TA_{3'}$ and $\Delta TA_{3\rightarrow 1}$ change. **Fig. 4 e, f** and **g** show the ideal cycles for various p_1 given fixed p_3, $TA_{3'}$ and $\Delta TA_{3\rightarrow 1}$. The amount of CO_2 captured in process 3'i→1 and monitored by the flow meter and the CO_2 sensor is around 50 mL, which translates to 0.21 M $\Delta DIC_{flow,3'i\rightarrow 1}$, assuming T = 293 K and p = 1 bar, across all different p_1 values (**Fig. 4a**). This similarity is consistent with the ideal cycle behavior, illustrated in **Fig. 4 c** and the alignment of measured $\Delta DIC_{flow,3'i\rightarrow 1}$ with the theoretical $\Delta DIC_{TA-eq,3'\rightarrow 1}$ *vs.* pCO_2 curve. The similar amount of CO_2 captured and released, i.e. $\Delta DIC_{flow,3'i\rightarrow 1}$ and $\Delta DIC_{flow,1'\rightarrow 3}$, is caused by the coincidental resemblance of the slopes of the two-stage deacidification+CO_2 invasion and the two-stage acidification+CO_2 outgassing processes under the experimental conditions (**Fig. 4 c**). The agreement of $\Delta DIC_{TA-eq,3'\rightarrow 1}$ *vs.* pCO_2 and $\Delta DIC_{TA-eq,1'\rightarrow 3}$ *vs.* pCO_2 curves at high p_1 values also corroborates the flow measurements. If the p_1 were 0.4 mbar instead, the deacidification and acidification processes would have significantly different slopes so the amounts of CO_2 captured during deacidification and released during acidification would be different, as shown in **Supplementary Figure 1 e**.

Here we plot the ideal cycles for varying $TA_{3'}$ and fixed $p_1 = 0.4$ mbar, p_3 = 1 bar and $\Delta TA_{3\rightarrow 1}$ = 0.21 M (**Supplementary Figure 1**). The plots illustrate the effect of varying $TA_{3'}$ on $w_{\mathrm{cycle,ideal}}$, $\Delta DIC_{\mathrm{TA-eq}}$, and $\overline{w}_{ideal}$.

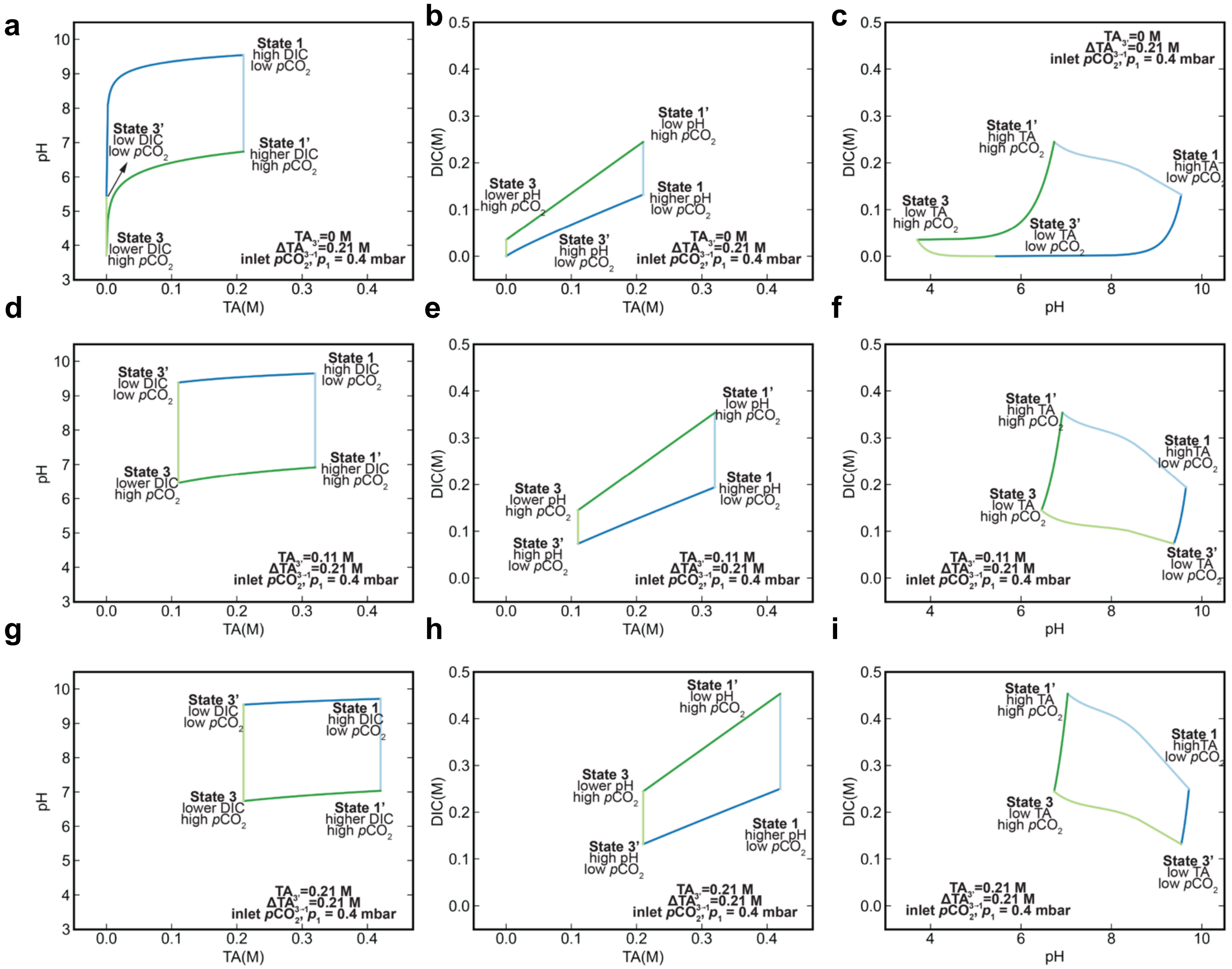

Supplementary Figure 1| Ideal cycles constructed using p_1 = 0.4 mbar and p_3 = 1 bar, $\Delta TA_{3\rightarrow 1}$ = 0.21 M and varying $TA_{3'}$. a-c, $TA_{3'}$ = 0.0 M; **d-f**, $TA_{3'}$ = 0.11 M and **g-i**, $TA_{3'}$ = 0.21 M;

The area enclosed by the cycles in **Supplementary Figure 1a, d** and **g**, is proportional to the ideal cycle work for the cycles with fixed p_1= 0.4 mbar, p_3 =1 bar, $\Delta TA_{3\rightarrow 1}$ = 0.21 M and $TA_{3'}$ being 0.0, 0.11 and 0.21 M, respectively. The area roughly stays the same as $TA_{3'}$ increases, but $\Delta DIC_{\mathrm{TA-eq},3\rightarrow 1}$ shrinks significantly, as shown in **Supplementary Figure 1b, e** and **h**. As a result, $\overline{w}_{ideal}$ increases as $TA_{3'}$ increases. With $TA_{3'}$ being 0.0, 0.11 and 0.21 M, the ideal cycle work is 3.58, 3.40 and 3.31 kJ L^{-1}, respectively, $\Delta DIC_{\mathrm{TA-eq},3\rightarrow 1}$ is 0.097, 0.049 and 0.005 M, respectively and the resulting $\overline{w}_{ideal}$ is 37.02, 69.31 and 661.9 kJ mol_{CO2}^{-1}, respectively.

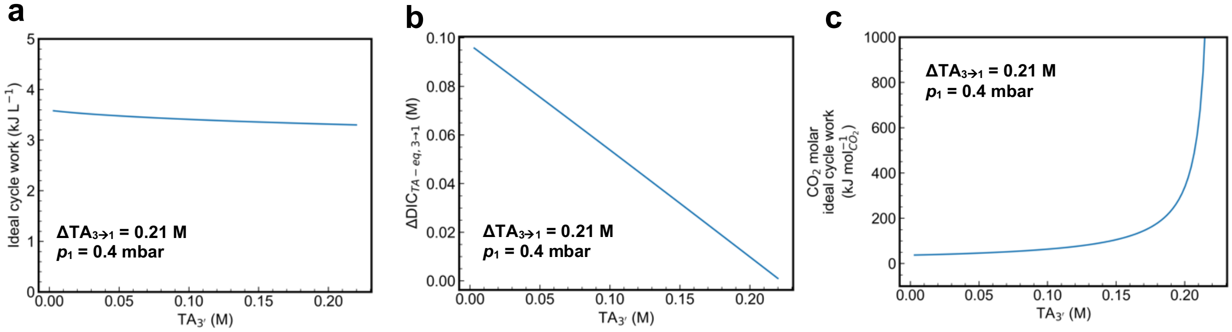

Supplementary Figure 2| Dependence of (a) $w_{\text{cycle,ideal}}$, (b) $\Delta DIC_{\text{TA−pH},3\to1}$ (c) and $\overline{w}_{ideal}$ on $TA_{3'}$ in the ideal cycles with p_1 = 0.4 mbar, and p_3 = 1 bar, $\Delta TA_{3\to1}$= 0.21 M.

Supplementary Figure 2 shows the dependence of $w_{\text{cycle,ideal}}$, $\Delta DIC_{\text{TA−eq},3\to1}$ and $\overline{w}_{ideal}$ on $TA_{3'}$. Again, $\overline{w}_{ideal}$ increases with increasing $TA_{3'}$ because of the linearly decreasing $\Delta DIC_{\text{TA−pH},3\to1}$ in the denominator of eq. S11.

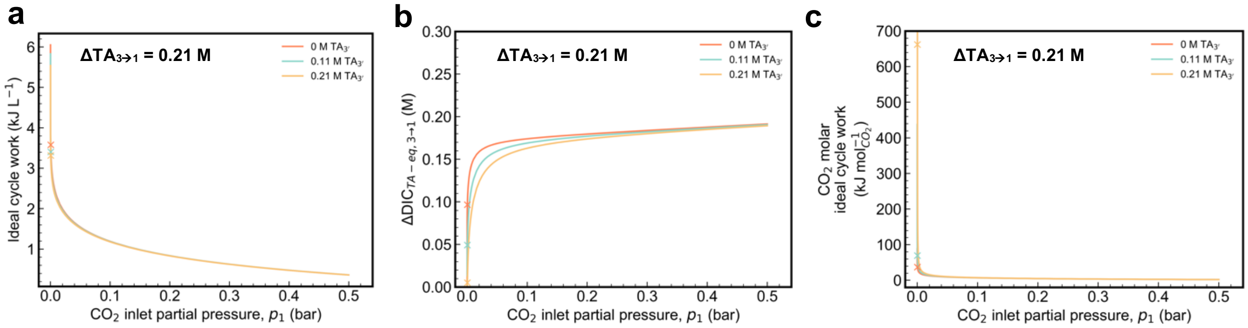

Supplementary Figure 3| Dependence of (a) $w_{\text{cycle,ideal}}$, (b) $\Delta DIC_{\text{TA−eq},3\to1}$ and (c) $\overline{w}_{ideal}$ on p_1 in the ideal cycles with $\Delta TA_{3\to1}$ = 0.21 M and $TA_{3'}$ = 0.0, 0.11 and 0.21 M. The 'x' marks indicate p_1 = 0.4 mbar.

Supplementary Figure 3 shows the dependence of $w_{\text{cycle,ideal}}$, $\Delta DIC_{\text{TA−eq},3\to1}$ and $\overline{w}_{ideal}$ on p_1. $w_{\text{cycle,ideal}}$ decreases as p_1 increases because of lower negolyte pH, hence cell potential, during deacidification, but the curves are almost identical for different $TA_{3'}$ values. The $\Delta DIC_{\text{TA−eq},3\to1}$ vs. p_1 curves for different $TA_{3'}$ are similar for large pCO_2 values, but significantly different at low p_1. Therefore, to keep $\overline{w}_{ideal}$ low for any inlet pressure p_1, high $TA_{3'}$ should be avoided.

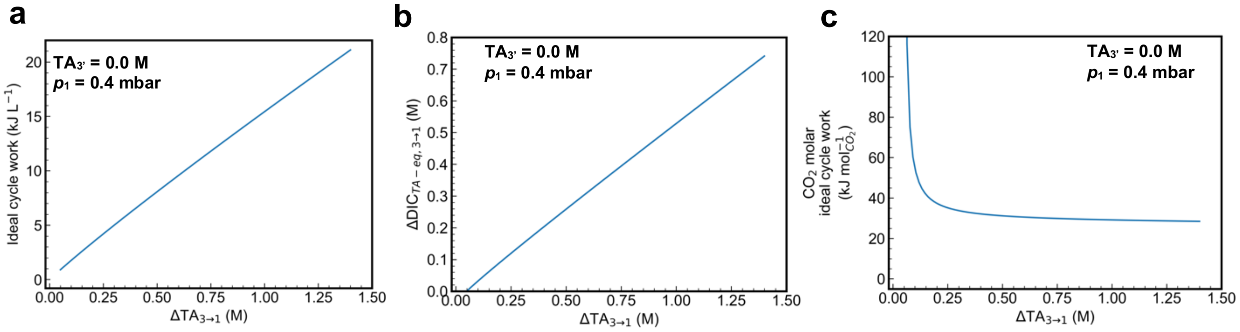

Supplementary Figure 4| Dependence of (a) $w_{\text{cycle,ideal}}$, (b) $\Delta DIC_{\text{TA−eq},3\to1}$ and (c) and $\overline{w}_{ideal}$ on $\Delta TA_{3\to1}$ in the ideal cycles with p_1 = 0.4 mbar, p_3 = 1 bar and $TA_{3'}$ = 0.0 M.

The last parameter that affects $w_{\text{cycle,ideal}}$, $\Delta DIC_{\text{TA−eq},3\to1}$ and $\overline{w}_{ideal}$ is $\Delta TA_{3\to1}$, which is equivalent to twice the amount of DSPZ concentration. DSPZ has a solubility of 0.7 M in 1 M KCl or 1 M

KOH aqueous solution, so the largest $\Delta TA_{3\rightarrow 1}$ it can induce is 1.4 M. **Supplementary Figure 4** shows the dependence of $w_{\text{cycle,ideal}}$, $\Delta DIC_{TA-eq,3\rightarrow 1}$ and $\bar{w}_{ideal}$ on $\Delta TA_{3\rightarrow 1}$ for cycles with $TA_{3'}$ = 0.0 M, p_1 = 0.4 mbar and p_3 = 1 bar. Not surprisingly, $w_{\text{cycle,ideal}}$ and $\Delta DIC_{TA-eq,3\rightarrow 1}$ increase with $\Delta TA_{3\rightarrow 1}$ but the difference in their increase rate causes $\bar{w}_{ideal}$ to decrease with larger $\Delta TA_{3\rightarrow 1}$. At 1.4 M $\Delta TA_{3\rightarrow 1}$, $\bar{w}_{ideal}$ is 28.45 kJ mol_{CO2}^{-1}, which is 23% lower than 37.02 kJ mol_{CO2}^{-1} for 0.21 M $\Delta TA_{3\rightarrow 1}$. Assuming that similar second law efficiency holds for experimental cycles with different $\Delta TA_{3\rightarrow 1}$ and same p_1, p_3 and $TA_{3'}$, using higher concentration of DSPZ can further decrease the cycle work for CO_2 separation from 72.8 kJ $mol_{CO2}^{-1}$to 64.2 kJ mol_{CO2}^{-1} at 5 mA cm^{-2}. With the same reasoning, if a PCET molecule that undergoes 2-e^- transfer and has 10 M solubility is developed, the ideal cycle work could be as low as 24 kJ mol_{CO2}^{-1}, leading to an actual cycle work of 59.8 kJ mol_{CO2}^{-1}.

2 Cycle Data

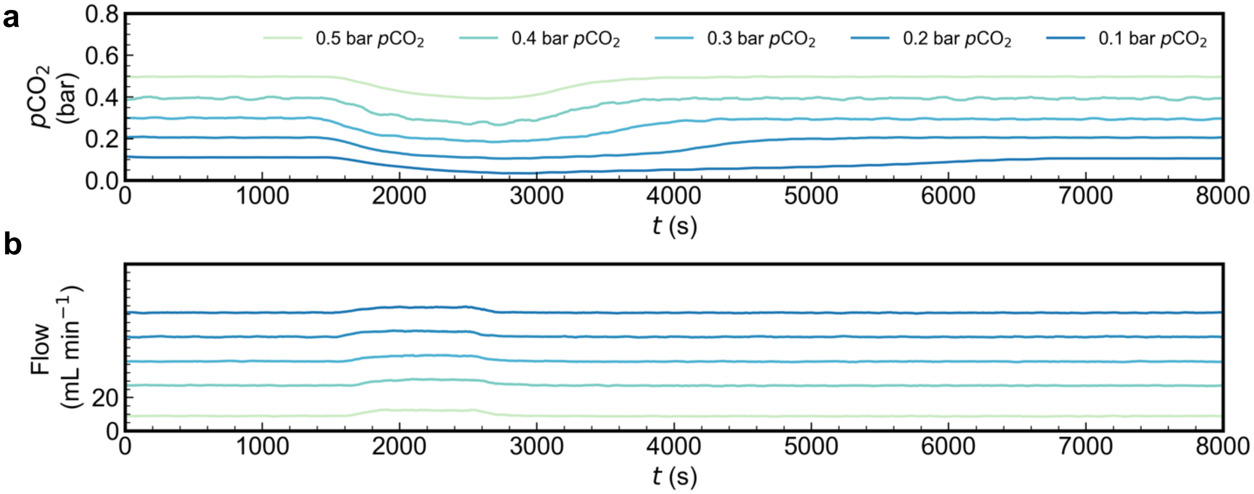

Supplementary Figure 5| Duration of the CO_2 capture and release processes in the cycles with 40 mA cm^{-2} current density. a, Downstream pCO_2 of one capture half cycle for each of the inlet pCO_2 conditions. **b,** Filtered total gas flow rate of one outgassing half cycle for each of the inlet pCO_2 conditions. All the processes look identical because of the same exit condition, so an arbitrary offset is added to differentiate the curves.

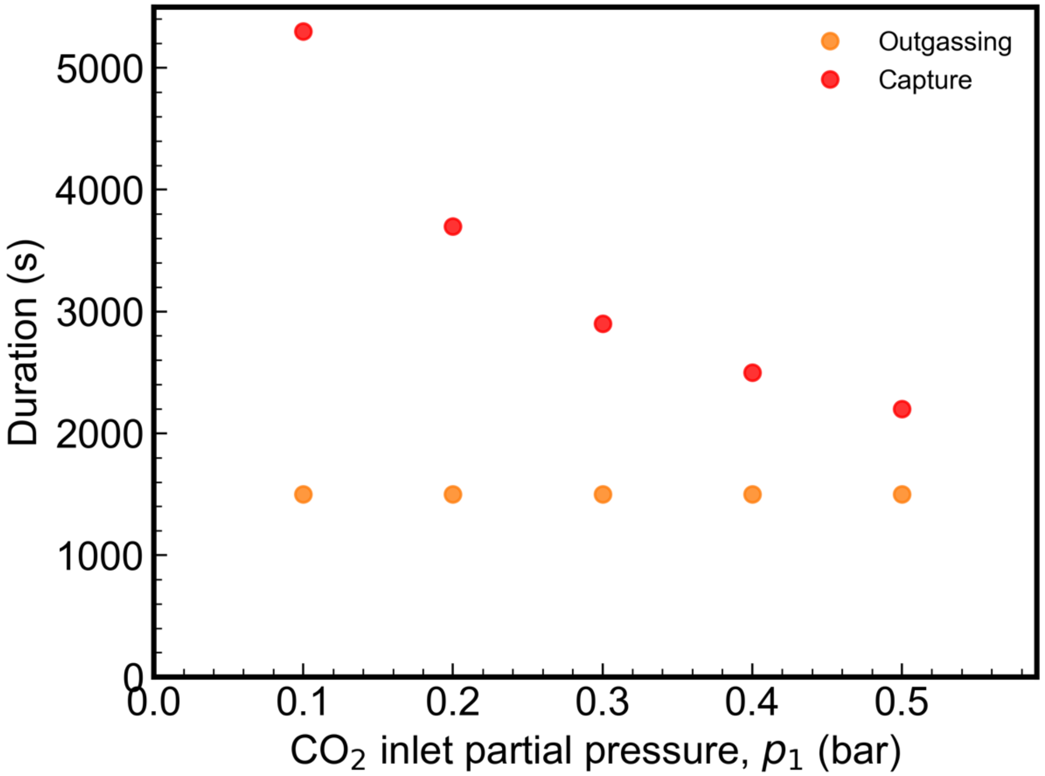

Supplementary Figure 6| Capture and outgassing durations extracted from Supplementary Figure 5 a and b.

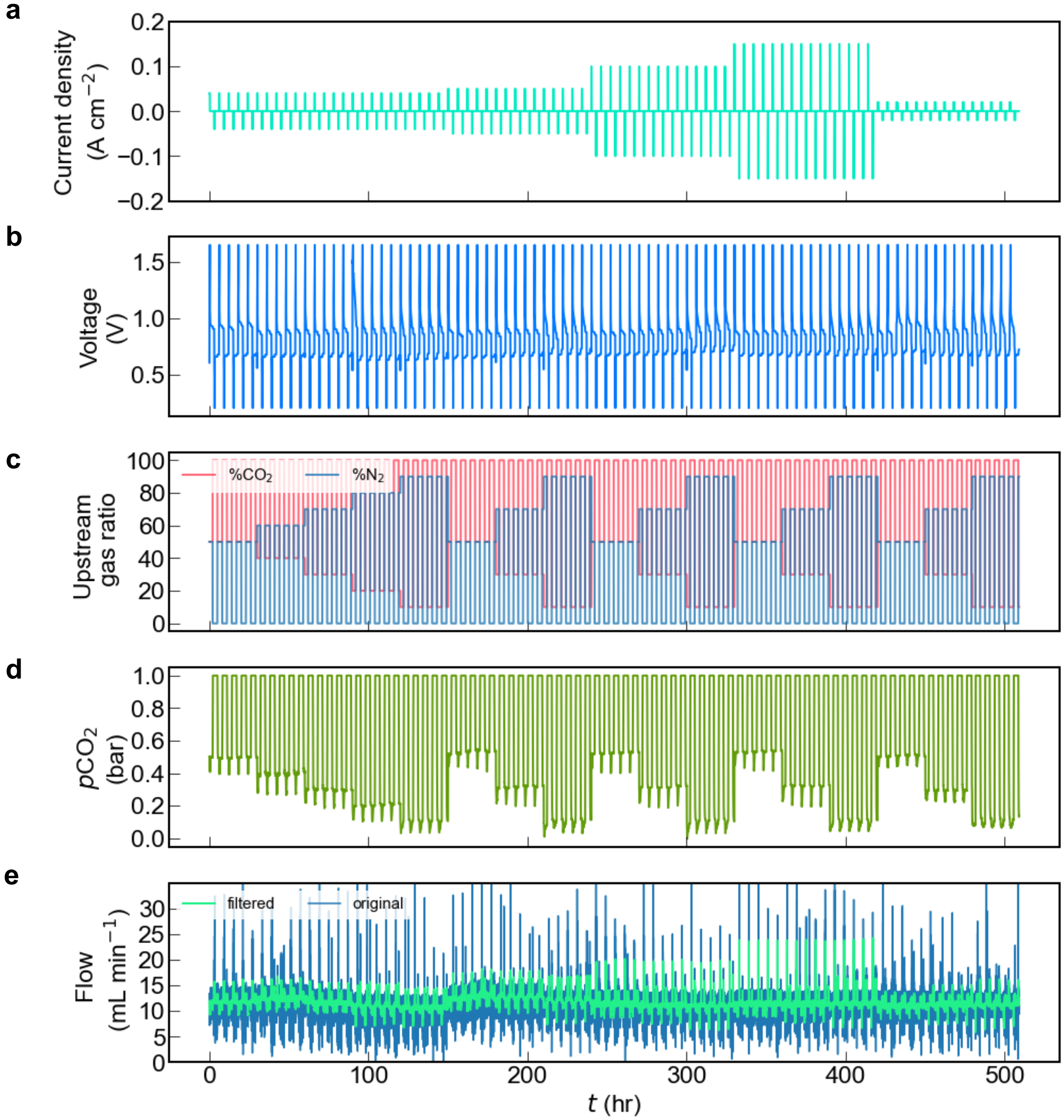

Supplementary Figure 7| Eighty-five CO_2 concentrating cycles with varying inlet pCO$_2$ and current densities. These are the raw data for Fig. 5. Same cell was used as in **Fig 2**. Liquid pumping rate is 50 mL min^{-1} for all the cycles. Note that, the pH measurements for high current densities are inaccurate, as the pH should never be able to reach pH > 14 for 0.11 M DSPZ. **a**, Current density. **b**, Voltage. **c**, N_2 and CO_2 percentage in the upstream source gas, controlled by mass flow controllers. **d**, CO_2 partial pressure. **e**, Total gas flow rate.

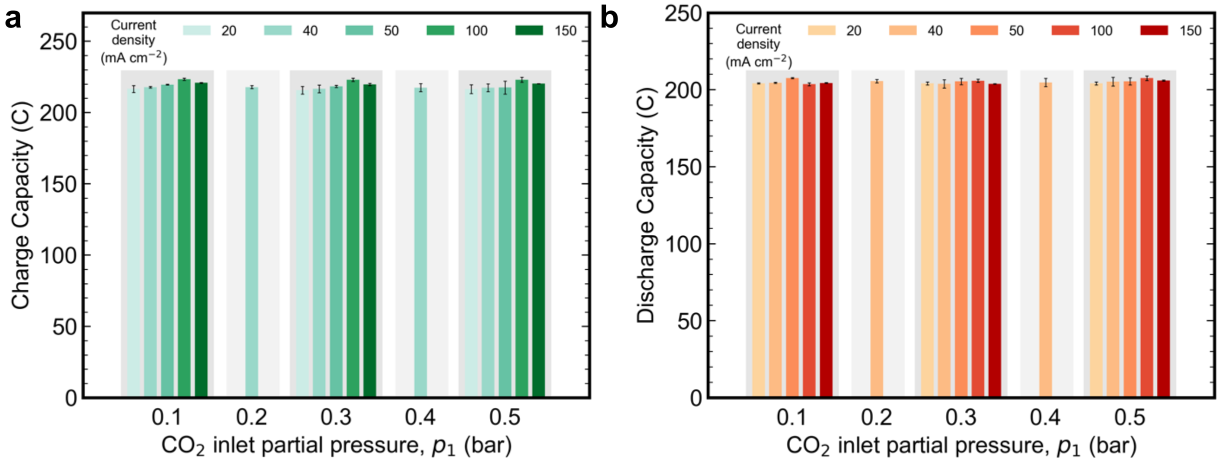

Supplementary Figure 8| Charge (a) and discharge (b) capacities of each experimental condition. In both a and b the horizontal axis is categorical, each shadowed region refers to a single p_1 value. The error bars refer to standard deviation.

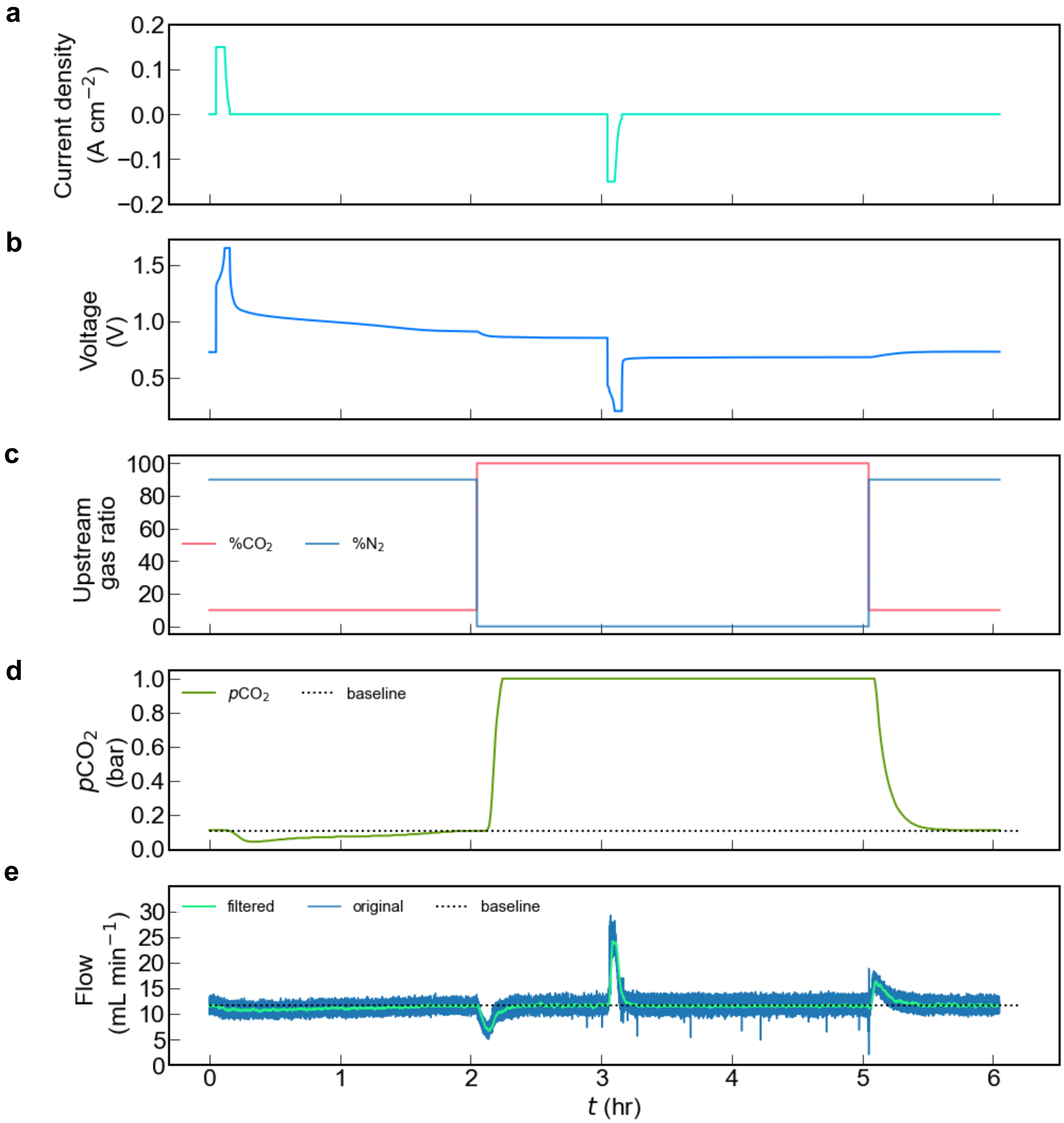

Supplementary Figure 9| One CO_2 concentrating cycle from Supplementary Figure 7 with 0.1 bar inlet pCO_2 and 1 bar exit pCO_2 at 150 mA cm^{-2}. Note that, the pH measurements are not shown because of an auxiliary port impedance issue present in the potentiostat hardware/software, so it is invalid to extract DIC_{TA-pH} for this condition. However, DIC_{TA-eq} can be extracted because sufficient gas-solution is reached, as demonstrated by fact that the *p*CO2 and flow curves return to their baselines after CO_2 invasion and outgassing. **a**, Voltage profile. **b**, Current density. **c**, N_2 and CO_2 percentage in the upstream source gas, controlled by MFC. **d**, CO_2 partial pressure. **e**, Total gas flow rate.

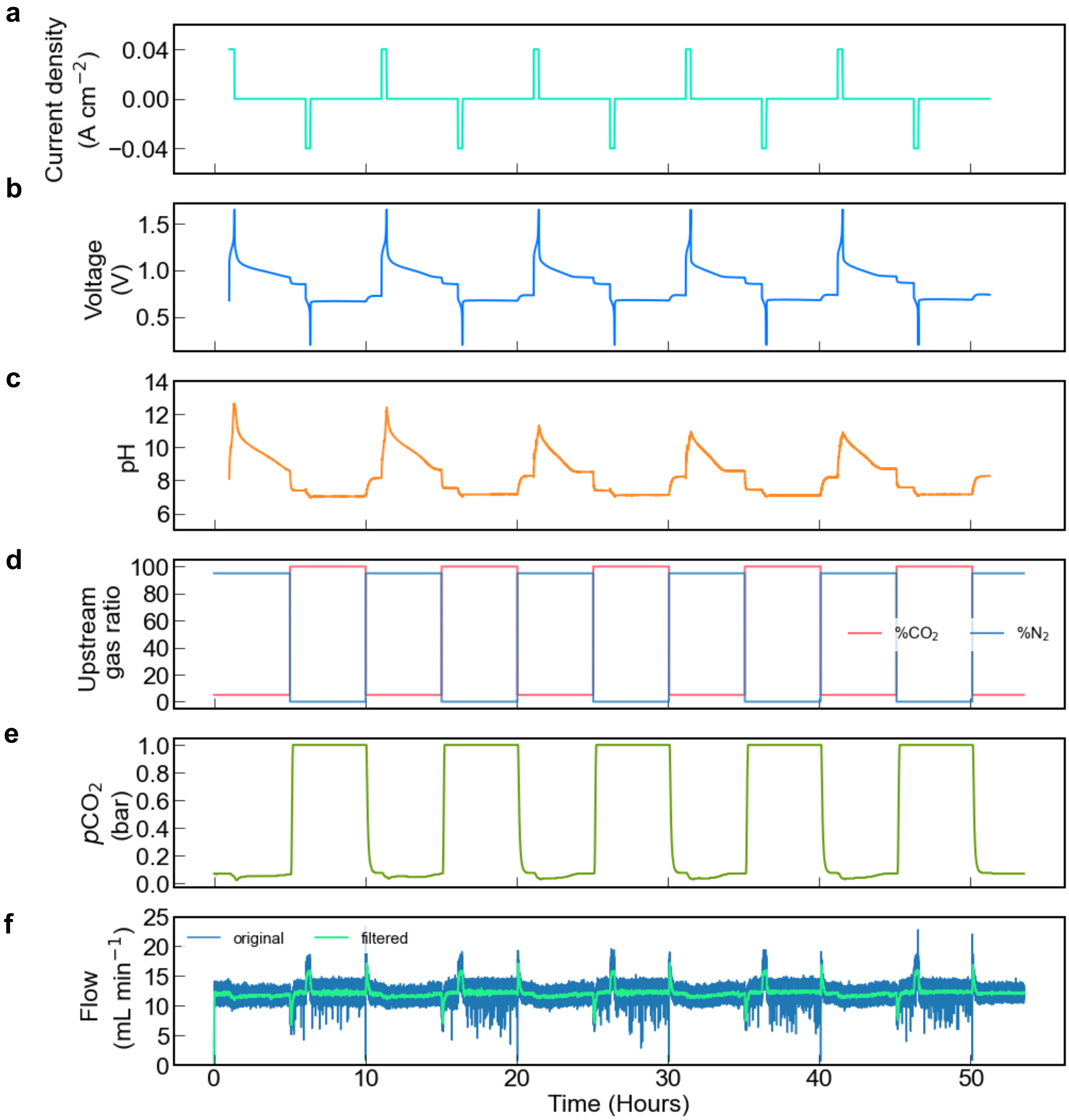

Supplementary Figure 10| Five CO_2 concentrating cycles with 0.05 bar inlet pCO_2 and 1 bar exit pCO_2 at 40 mA cm^{-2}. Same cell was used as in **Fig. 2**. Fresh negolyte and posolyte were used. The liquid pumping rate is 150 mL min^{-1}, which is 50% faster than for capture at higher inlet pressure. **a**, Voltage profile. **b**, Current density. **c**, pH of the negolyte. **d**, N_2 and CO_2 percentage in the upstream source gas, controlled by mass flow controllers. **e**, CO_2 partial pressure. **f**, Total gas flow rate.

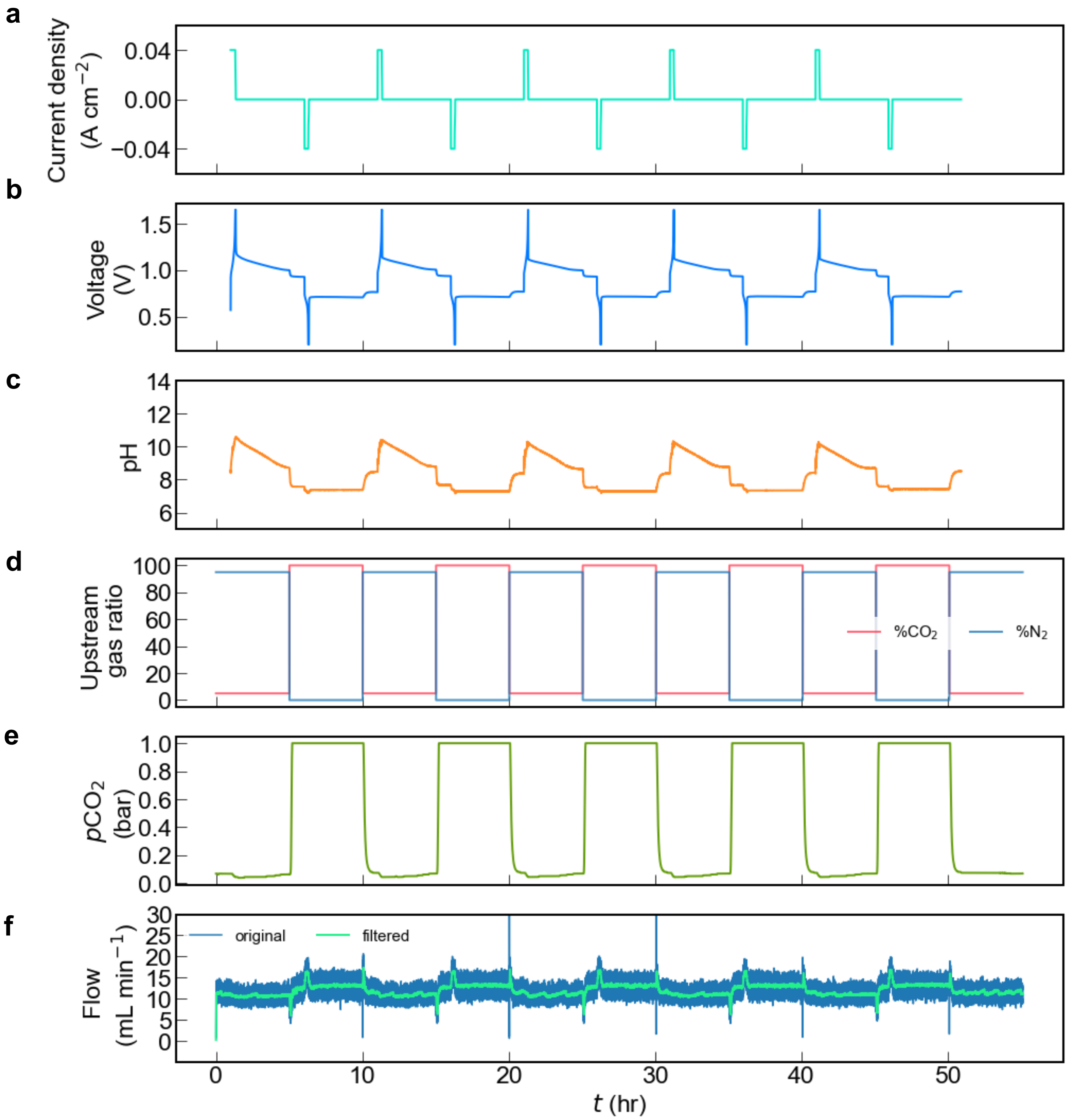

Supplementary Figure 11| Post-electrochemical rebalancing CO_2 capture with 0.05 bar inlet pCO_2 and 1 bar exit pCO_2 at 40 mA cm^{-2}. Same cell was used as in **Fig.2**. Same posolyte and negolyte as in **Supplementary Figure 10** were used. The liquid pumping rate is 150 mL min^{-1}. **a**, Voltage profile. **b**, Current density. **c**, pH of the negolyte. **d**, N_2 and CO_2 percentage in the upstream source gas, controlled by mass flow controllers. **e**, CO_2 partial pressure. **f**, Total gas flow rate. The system has the same carbon capture/release capability after the post-electrochemical rebalancing.

3 Non-Linear Fit of Molar Cycle Work With Tafel Model

This section interprets the origin of the non-linear trend of the molar cycle work and describes in detail the Tafel model that fits the non-linear behavior.

The molar cycle work values shown in **Fig. 5e** and **f** are composed of ideal molar cycle work at given inlet CO_2 partial pressure p_1 and $TA_{3',i}$, and work loss associated with cell inefficiencies, i.e.

$$\bar{w} = \bar{w}_{ideal} + \bar{w}_{cell}; \quad \text{(S13)}$$

where $\bar{w}$ is the experimental molar cycle work (eq. 4 in the method section of main text), $\bar{w}_{ideal}$ is the ideal molar cycle work (eq. S12) and $\bar{w}_{cell}$ is the molar cycle work loss associated with cell inefficiency beyond the irreversibilities inherent in the ideal cycle. $\bar{w}_{cell}$ is expressed as:

$$\bar{w}_{cell} = \frac{q(\eta_{ohmic} + \eta_{et} + \eta_{mt})}{\Delta DIC_{3\rightarrow 1} V} \quad \text{(S14)}$$

where q is the charge passed in a half-cycle; V is the electrolyte volume; $\Delta DIC_{3\rightarrow 1}$ is the DIC difference between state 1 and state 3; η_{ohmic} is the ohmic overpotential; η_{et} is the electron transfer, or sometimes called activation or kinetic, overpotential; and η_{mt} is the mass transport overpotential. η_{ohmic} arises from ohmic resistance, such as membrane resistance, and is proportional to the applied current, i.e.

$$\eta_{ohmic} = i r_{ohmic} \quad \text{(S15)}$$

where i is the applied current density and r_{ohmic} is the area specific ohmic resistance, which is 1.4 Ω cm^2, measured by electrochemical impedance spectrometry, in the cell we used. We also assume that the losses caused by under-pressure during CO_2 invasion and over-pressure during CO_2 outgassing [ref Jin *et al.*] are proportional to current and consequently are included in the ohmic overpotential term; we find that adding 0.1 Ω cm^2 for these effects permits us to fit the data reasonably well by the process described below. η_{ohmic} is the sum of the absolute values of the overpotentials during acidification and deacidification. Because η_{ohmic} is proportional to current and η_{mt} is usually negligible within a flow battery system at modest current densities like our case,[3] we interpret the cause of the non-linear behavior in **Fig. 5e** and **5f** to be η_{et}. The electron transfer overpotential consists of a deacidification and an acidification contribution, i.e:

$$\eta_{et} = \eta_{deacidification} - \eta_{acidification} \quad \text{(S16)}$$

where $\eta_{deacidification}$ is the electron transfer overpotential during the deacidification process, which has a positive value, and $\eta_{acidification}$ is the electron transfer overpotential during the acidification process, which has a negative value, as discussed below. Both deacidification and acidification reactions involve an anodic half reaction and a cathodic half reaction, so each of $\eta_{deacidification}$ and $\eta_{acidification}$ has an anodic and a cathodic contribution. In our cell, the participating half reactions in deacidification are:

$$\text{Anodic: } Fe(CN)_6^{4-} \rightarrow Fe(CN)_6^{3-} + e^- \quad \text{(S17)}$$

$$\text{Cathodic: } DSPZ + 2e^- + 2H_2O \rightarrow DSPZH_2 + 2OH^- \quad \text{(S18)}$$

Assuming that the backward reactions in eq. S17 and S18 are negligible, which is a good approximation when the electron transfer overpotential is large (>118 mV), $\eta_{deacidification}$ is expressed as:

$$\eta_{deacidification} = \eta_{anodic,Fe} - \eta_{cathodic,DSPZ} \quad \text{(S19)}$$

where $\eta_{anodic,Fe}$ and $\eta_{cathodic,DSPZ}$ represent, respectively, the overpotentials from the electron transfer process of the anodic half reaction in the $Fe(CN)_6^{4-}/Fe(CN)_6^{3-}$ posolyte, i.e. eq. S17, and the cathodic half reaction in the DSPZ/$DSPZH_2$ negolyte, i.e. eq. S18.

Similarly, the participating half reactions in acidification are:

$$\text{Cathodic: } Fe(CN)_6^{3-} + e^- \rightarrow Fe(CN)_6^{4-} \quad \text{(S20)}$$

$$\text{Anodic: } DSPZH_2 + 2OH^- \rightarrow DSPZ + 2e^- + 2H_2O \quad \text{(S21)}$$

Again, assuming the backward reactions in eq. S20 and S21 are negligible, $\eta_{acidification}$ is expressed as

$$\eta_{acidification} = \eta_{cathodic,Fe} - \eta_{anodic,DSPZ} \quad \text{(S22)}$$

where $\eta_{cathodic,Fe}$ and $\eta_{anodic,DSPZ}$ represent, respectively, the overpotentials from the cathodic and anodic half reactions. These $\eta_{cathodic}$ and η_{anodic} values are well modelled by the Tafel equations for the large overpotential region[4]:

$$\eta_{anodic} = \frac{RT}{(1-\alpha)nF} \ln \frac{|i|}{i_0} \quad \text{(S23)}$$

$$\eta_{cathodic} = \frac{RT}{\alpha nF} \ln \frac{i_0}{|i|} \quad \text{(S24)}$$

Substituting eq. S23 and S24 into eq. S22, S19 and S16, we obtain the expression of η_{et} as:

$$\eta_{et} = \frac{RT}{(1-\alpha_{Fe})1F} \ln \frac{|i|}{i_{0,Fe}} - \frac{RT}{\alpha_{DSPZ}2F} \ln \frac{i_{0,DSPZ}}{|i|} - \frac{RT}{\alpha_{Fe}1F} \ln \frac{i_{0,Fe}}{|i|} + \frac{RT}{(1-\alpha_{DSPZ})2F} \ln \frac{|i|}{i_{0,DSPZ}} \quad \text{(S25)}$$

Because eq. S25 describes the overpotential associated with a composite of four electron transfer processes, it is difficult to disentangle the contribution from each process. Therefore, for simplicity, we modelled it with the constraint that all i_0 values are equal and we set both α_{Fe} and α_{DSPZ} to be 0.5, supported by the symmetric shapes of the cyclic voltammograms of both species. [2,5] So eq. S25 is simplified to:

$$\eta_{et} = \frac{6RT}{F} \ln \frac{|i|}{i_0} \quad \text{(S26)}$$

The inputs for eq. S26 are η_{et} calculated from eq. S11-13 and the applied current density, and a non-linear least squares method is applied to fit for i_0

For high partial pressure capture data presented in Fig. 5e, $\overline{w}_{ideal}$ values are calculated using eq. S1-12 with p_3 = 1 bar and $TA_{3'i}$ = 0.11 M, and the resulting values are 6.85, 3.4 and 1.88 kJ $molCO_2^{-1}$ for p_1 = 0.1, 0.3 and 0.5 bar, respectively. Other parameters q and $\Delta DIC_{3\to 1}$ are available from **Supplementary Figure 8** and Fig. 5b and V is 10 mL. The fitted curves are shown in **Supplementary Figure 12**.

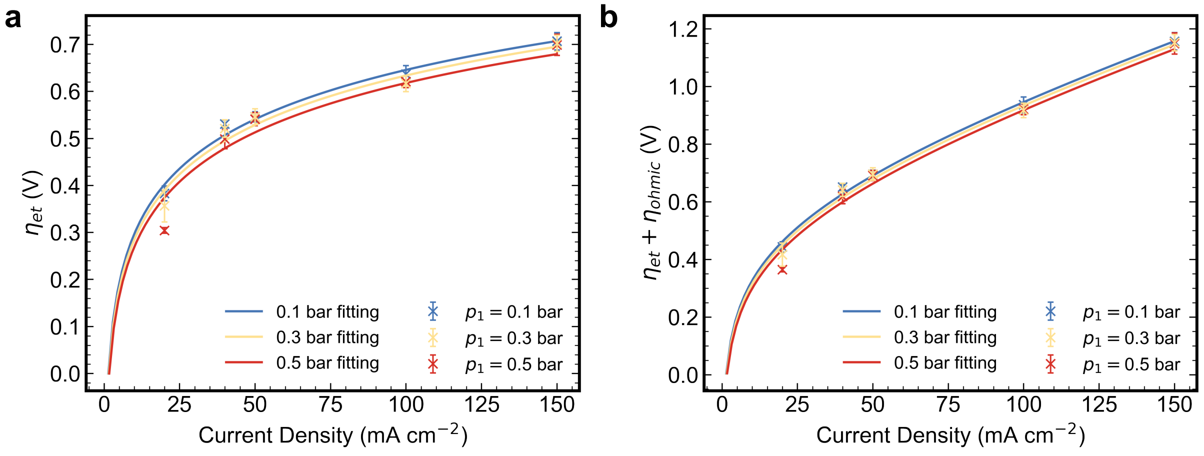

Supplementary Figure 12| The fitted curves η_{et} at p_1 = 0.1, 0.3 and 0.5 bar using the Tafel model. (a) η_{et} only and (b) the sum of η_{et} and η_{ohmic}.

The fitted i_0 values are 1.4, 1.5 and 1.7 mA cm^{-2} for p_1 = 0.1, 0.3 and 0.5 bar, respectively. The fitting can be improved with more data points taken at different current densities and the assumption of $\alpha_{,Fe} = \alpha_{,DSPZ} = 0.5$ relaxed.

For p_1 = 0.4 mbar, the molar cycle work taken from figure 5f and $\overline{w}_{ideal}$ at $\Delta TA_{3\to 1}$ = 0.21 M is 68.7 and 36.9 kJ $molCO_2^{-1}$ for $TA_{3'i}$ = 0.11 and 0.0 M, respectively, as explained in the "Ideal Cycle Work" section in the supporting information. **Supplementary Figure 13** shows the fitted curves.

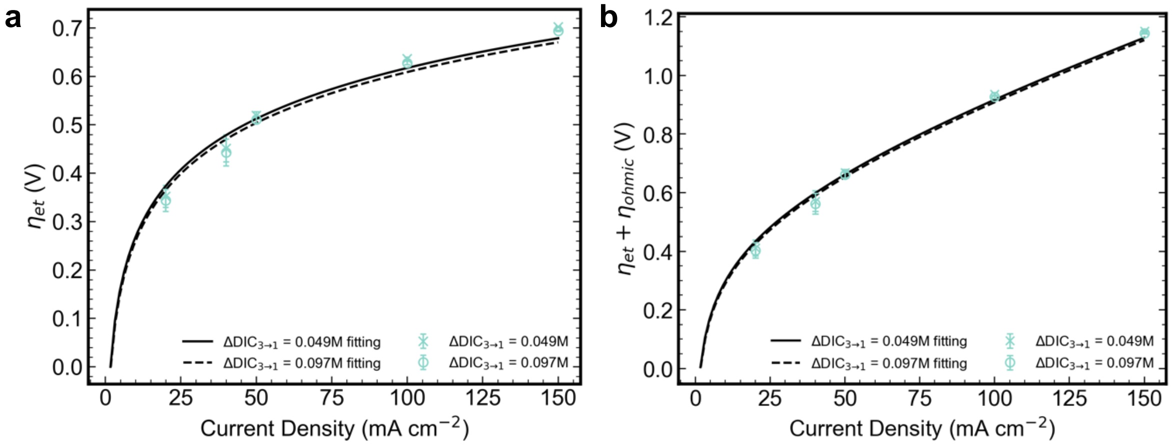

Supplementary Figure 13| The fitted curves η_{et} at p_1 = 0.4 mbar using the Tafel model. $\Delta DIC_{3\to1}$ = 0.049 M when TA3'i = 0.11 M and $\Delta DIC_{3\to1}$ = 0.097 M when TA3'i = 0.0 M. (a) η_{et} only and (b) the sum of η_{et} and η_{ohmic}.

The fitted i_0 values are 1.7 and 1.8 mA cm^{-2} for $TA_{3'i}$ = 0.11 and 0.0 M, respectively. The slightly higher value of η_{et} and the consequently lower value of i_0 at $TA_{3'i}$ = 0.11 M than at 0.0 M is caused by the fact that $w_{cycle,ideal}$ is slightly smaller at $TA_{3'i}$ = 0.11 M, being 3.39 J L^{-1}, versus 3.57 J L^{-1} at $TA_{3'i}$ = 0.0 M (**Supplementary Figure 2** a). The similar behavior for the two conditions of η_{et} plus η_{ohmic} suggests the dramatic difference in molar cycle work in Fig. 5f is caused by the difference in $\Delta DIC_{3\to1}$ and $\overline{w}_{ideal}$ (eq. S11-12).

4 More on Electrochemical Rebalancing

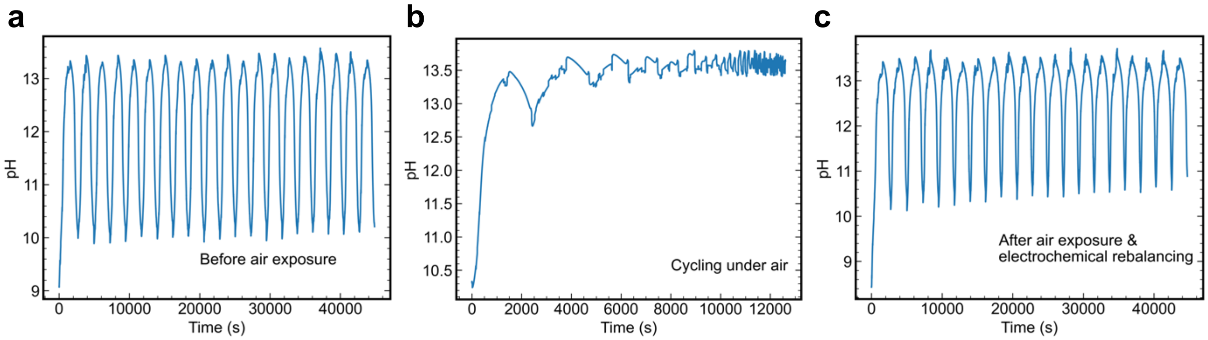

Supplementary Figure 14| pH of the negolyte during cycles before air exposure (a), under air (b) and after electrochemical rebalancing (c), respectively. pH drifts up because of oxygen presence.

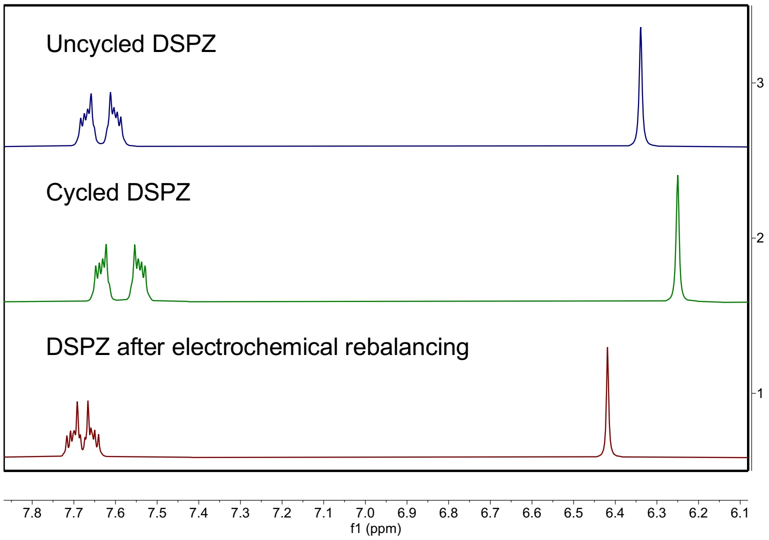

Supplementary Figure 15| NMR spectra of aromatic region of (top) uncycled DSPZ, (middle) DSPZ after cycling under air, and (bottom) DSPZ after electrochemical rebalancing. No new peaks in the aromatic region were observed. The slight peak shifts were caused by concentration and pH differences.

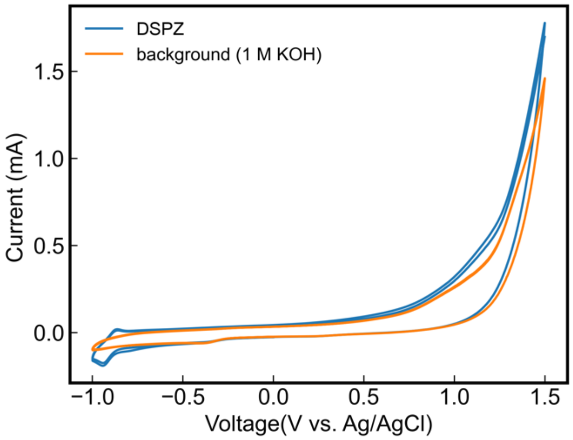

Supplementary Figure 16| Cyclic voltammetry of DSPZ and 1 M KOH background. No additional peak was observed for DSPZ during the oxidative scan, indicating absence of side reactions.

Fig. 1b lists all the reactions related to carbon capture in our system. When all reactions involving CO_2 are removed, the system is the same as an aqueous organic redox flow battery (AORFB).[6-8] The electrochemical rebalancing method is also applicable to AORFB when there is an oxygen leakage. **Fig. 6** demonstrates the application of the electrochemical rebalancing method in an AORFB and carbon capture flow cell, which both are organic PCET systems that have pH swing ranging from neutral to basic. **Supplementary Figure 15** shows that there are no new peaks generated in the aromatic region in the NMR spectra, indicating the absence of side reactions during electrochemical rebalancing. Based on the result in **Fig. 6**, the electrochemical rebalancing of a completely out-of-balance cell consumes roughly three times the cycle work of a single carbon capture cycle at 40 mA cm^{-2}. Therefore, performing one rebalancing step per 30 cycles increases the cycle work per cycle by 10%, which is approximately an acceptable upper limit. Therefore, each cycle is allowed to have 3.3% of the reduced PCET molecule oxidized by oxygen. We also calculated a target capture duration to be 8 hours for DAC (we used equations in Stolaroff *et al.*[9]

and assumed 2 M KOH. The calculation indicates that an 8-hour capture period is achievable with a 2mm thick sorbent bed). So the max-tolerable oxidation rate is 0.4% hr^{-1} under air. Note that the energy cost of the rebalancing step will be reduced if a smaller current density is applied or an electrode that facilitates oxygen evolution reaction is used. As a result, the oxidation rate requirement on the PCET molecule may be loosened.

Here we suggest that the electrochemical rebalancing method also applies to other aqueous based electrochemical systems, including organic and inorganic, PCET or non-PCET, acidic or basic, dissolved or solid redox active materials. If no side reaction is triggered by the oxidative voltage, which is the case for DSPZ as shown in **Supplementary Figure 16**, the electrochemical rebalancing method can be applied. Here are several examples.

Organic Non-PCET system in neutral aqueous solution: $Fe(CN)_6$ (posolyte) | Viologen (negolyte) Flow Battery

When the viologen-based redox flow battery[5,10,11] is charged: oxygen can chemically oxidize the reduced viologen to the oxidized state, accumulating hydroxide in the negolyte, leading to the negolyte to discharged state and the posolyte active species maintaining the oxidized state. Because the redox active core of viologens have two positive charges, we denote their oxidized form as Vi^{2+} and the single-electron reduced form as Vi^{+}. The negolyte side is discharged when oxygen is present, i.e. :

$$\frac{1}{2}O_2 + 2\,Vi^+ + H_2O \rightarrow 2\,Vi^{2+} + 2\,OH^-$$

The electrochemical rebalancing method can remove the accumulated hydroxide, repelling O_2 in the negolyte reservoir:

$$2\,OH^- \rightarrow \frac{1}{2}O_2 + H_2O + 2\,e^-$$

During the electrochemical rebalancing process, the electrons are transferred to the posolyte side, which has accumulated $Fe(CN)_6^{3-}$, and eventually both negolyte and posolyte sides are recover the their initial composition, i.e. $Fe(CN)_6^{4-}$ in posolyte and Vi^{2+} in negolyte, rebalancing the system.

Inorganic Non-PCET system in strongly acidic aqueous solution: VO^{2+}/VO_2^{+} (posolyte) | V^{3+}/V^{2+} (negolyte) Flow Battery

When a vanadium redox flow battery[12] negolyte contains the charged form, i.e. V^{2+}: if oxygen diffuses into the negolyte, it can chemically oxidize V^{2+} to V^{3+}, and hydroxide is accumulated in the negolyte,

$$\frac{1}{2}\,O^2 + 2\,V^{2+} + H_2O \rightarrow 2\,V^{3+} + 2\,OH^-$$

The electrochemical rebalancing method can remove the accumulated hydroxide, repelling O_2:

$$2\,OH^- \rightarrow \frac{1}{2}O_2 + H_2O + 2\,e^-$$

Because the electrolyte of a vanadium redox flow battery is strongly acidic, the hydroxide is readily neutralized and forming water. Hence the oxidation reaction is the following:

$$\frac{1}{2}O_2 + 2V^{2+} + 2H^+ \rightarrow 2V^{3+} + H_2O$$

Therefore, instead of generating two hydroxides in the negolyte, the oxidation by oxygen reaction causes the loss of two protons. And the electrochemical rebalancing method in such scenario is as follows:

$$H_2O \rightarrow \frac{1}{2}O_2 + 2H^+ + 2e^-$$

During the electrochemical rebalancing process, the electrons are transferred to the posolyte side, which has accumulated the oxidized form VO^{2+}, through

$$2VO^{2+} + 2e^- \rightarrow 2VO_2^+$$

, and eventually both negolyte and posolyte sides are fully discharged (VO_2^+ in posolyte and V^{3+} in negolyte), thus rebalancing the system.

Inorganic Non-PCET system in basic aqueous solution: air (posolyte) | S_4^{2-}/S_4^{4-} (negolyte) Battery

When a sulfur-air flow battery[13] is charged: if oxygen diffuses into polysulfide negolyte, oxygen can chemically oxidize polysulfide, and hydroxide is accumulated in the negolyte,

$$\frac{1}{2}O_2 + 2S_4^{4-} + H_2O \rightarrow 2S_4^{2-} + 2OH^-$$

The electrochemical rebalancing method can remove the accumulated hydroxide, repelling O_2:

$$2OH^- \rightarrow \frac{1}{2}O_2 + H_2O + 2e^-$$

During the electrochemical rebalancing process, the electrons are transferred to the posolyte side externally, thus rebalancing the system.

Solid polyquinone Non-PCET system for carbon capture: $LiFePO_4$ (cathode) | Polyquinone (anode)

Liu et al.[14] demonstrated a solid quinone aqueous carbon capture system, where the cathode is $LiFePO_4$ and the anode is polyquinone (PAQ) tethered to a carbon electrode. The authors utilized a 20 molal LiTFSI aqueous solution to ensure that the reduced PAQ are deprotonated, i.e. PAQ^{2-}, which then binds with CO_2 to form PAQ-CO_2 adduct. Although the influence of oxygen in this system is rather small, but side reaction still happens and can cause long term imbalance (accumulation of oxidized cathode material and accumulated LiOH in the anode side).

When the anode is charged: oxygen can chemically oxidize the air-sensitive anode, and hydroxide is accumulated in the negolyte,

$$\frac{1}{2}O_2 + H_2O + PAQ^{2-} \rightarrow PAQ + 2OH^-$$

The electrochemical rebalancing method can remove the accumulated hydroxide, repelling O_2:

$$2OH^- \rightarrow \frac{1}{2}O_2 + H_2O + 2e^-$$

During the electrochemical rebalancing process, the electrons are transferred to the cathode side externally, eventually both anode and cathode are discharged, rebalancing the system.

5 **Synthesis**

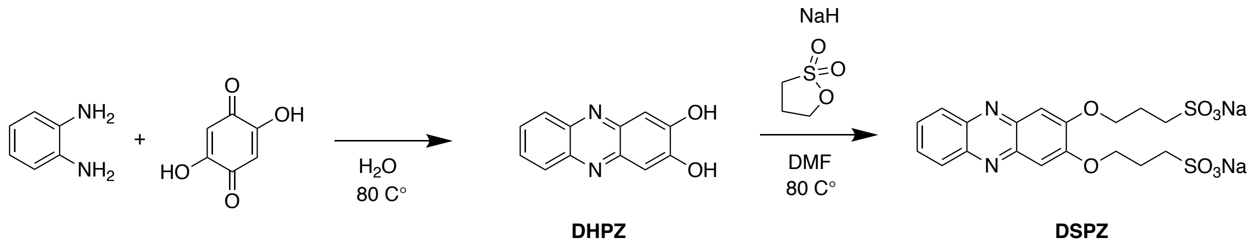

Supplementary Figure 17| Synthesis of DSPZ.

Benzene-1,2-diamine (1 equiv.) was mixed with 2,5-dihydroxycyclohexa-2,5-diene-1,4-dione (1.03 equiv.) in water to achieve 0.2 M benzene-1,2-diamine solution in a pressure vessel. The reaction mixture was refluxed at 80 °C and stirred overnight. The resulting slurry was filtered, and the black precipitate was crude product phenazine-2,3-diol (DHPZ). The black precipitate was then dissolved in 0.1 M KOH solution to make a 0.02 M DHPZ solution. The solution was filtered again and the filtrate was acidified with HCl solution until pH reached 7. Red precipitates formed and were filtered to give pure DHPZ (81% yield).

DHPZ (1 equiv.) was dissolved in DMF to make 0.05 M DHPZ solution. NaH (60 wt. % in mineral oil) (2.2 equiv. NaH) was added to the DHPZ solution under N_2. After all bubbles disappeared, 2.05 equiv. propane sultone was then added into the solution. The reaction mixture was stirred overnight at 80 °C to give a red slurry. The slurry was then cooled and filtered. The red precipitates were washed thoroughly with ethyl acetate to remove residual DMF. The final DSPZ products were red solids (65% yield)

Supplementary References

[1]R.N. Roy, L.N. Roy, K.M. Vogel, C. Portermoore, T. Pearson, C.E. Good, F.J. Millero, and D.M. Campbell, "The Dissociation-Constants of Carbonic-Acid in Seawater at Salinities 5 to 45 and Temperatures 0 °C to 45 °C", *Marine Chemistry* **44**, 249 (1993).

[2]S. Jin, M. Wu, R.G. Gordon, M.J. Aziz, and D.G. Kwabi, "pH Swing Cycle for CO_2 Capture Electrochemically Driven through Proton-Coupled Electron Transfer", *Energy & Environmental Science* **13**, 3706 (2020).

[3]Q. Chen, M.R. Gerhardt, and M.J. Aziz, "Dissection of the Voltage Losses of an Acidic Quinone Redox Flow Battery", *Journal of the Electrochemical Society* **164**, A1126 (2017).

[4]A.J. Bard and L.R. Faulkner, *Electrochemical Methods : Fundamentals and Applications*. (Wiley, New York, 2001).

[5]S. Jin, E.M. Fell, L. Vina-Lopez, Y. Jing, P.W. Michalak, R.G. Gordon, and M.J. Aziz, "Near Neutral pH Redox Flow Battery with Low Permeability and Long-Lifetime Phosphonated Viologen Active Species", *Advanced Energy Materials* **10**, 2000100 (2020).

[6]A. Hollas, X.L. Wei, V. Murugesan, Z.M. Nie, B. Li, D. Reed, J. Liu, V. Sprenkle, and W. Wang, "A Biomimetic High-Capacity Phenazine-Based Anolyte for Aqueous Organic Redox Flow Batteries", *Nature Energy* **3**, 508 (2018).

[7]S. Jin, Y. Jing, D.G. Kwabi, Y. Ji, L. Tong, D. De Porcellinis, M.A. Goulet, D.A. Pollack, R.G. Gordon, and M.J. Aziz, "A Water-Miscible Quinone Flow Battery with High Volumetric Capacity and Energy Density", *ACS Energy Letters* **4**, 1342 (2019).

[8]Y. Ji, M.A. Goulet, D.A. Pollack, D.G. Kwabi, S. Jin, D. Porcellinis, E.F. Kerr, R.G. Gordon, and M.J. Aziz, "A Phosphonate-Functionalized Quinone Redox Flow Battery at near-Neutral pH with Record Capacity Retention Rate", *Advanced Energy Materials* **9**, 1900039 (2019).

[9]J.K. Stolaroff, D.W. Keith, and G.V. Lowry, "Carbon Dioxide Capture from Atmospheric Air Using Sodium Hydroxide Spray", *Environ Sci Technol* **42**, 2728 (2008).

[10]E.S. Beh, D. De Porcellinis, R.L. Gracia, K.T. Xia, R.G. Gordon, and M.J. Aziz, "A Neutral pH Aqueous Organic-Organometallic Redox Flow Battery with Extremely High Capacity Retention", *ACS Energy Letters* **2**, 639 (2017).

[11]J. Luo, B. Hu, C. Debruler, Y.J. Bi, Y. Zhao, B. Yuan, M.W. Hu, W.D. Wu, and T.L. Liu, "Unprecedented Capacity and Stability of Ammonium Ferrocyanide Catholyte in Ph Neutral Aqueous Redox Flow Batteries", *Joule* **3**, 149 (2019).

[12]E. Mena, R. Lopez-Vizcaino, M. Millan, P. Canizares, J. Lobato, and M.A. Rodrigo, "Vanadium Redox Flow Batteries for the Storage of Electricity Produced in Wind Turbines", *International Journal of Energy Research* **42**, 720 (2018).

[13]Z. Li, M.S. Pan, L. Su, P.C. Tsai, A.F. Badel, J.M. Valle, S.L. Eiler, K. Xiang, F.R. Brushett, and Y.M. Chiang, "Air-Breathing Aqueous Sulfur Flow Battery for Ultralow-Cost Long-Duration Electrical Storage", *Joule* **1**, 306 (2017).

[14]Y. Liu, H.-Z. Ye, K.M. Diederichsen, T.V. Voorhis, and T.A. Hatton, "Electrochemically Mediated Carbon Dioxide Separation with Quinone Chemistry in Salt-Concentrated Aqueous Media", *Nature Communications* **11**, 2278 (2020).
